# Batch- and Composition-Controlled Reanalysis of Bulk and Single-Nucleus Transcriptomes Reveals Co-Enrichment of Glial Complement and of Translational Signatures in Parkinson’s Disease and Amyotrophic Lateral Sclerosis

**DOI:** 10.3390/ijms27188011

**Published:** 2026-09-09

**Authors:** Chaeyun Jung

**Affiliations:** Department of Experimental and Clinical Pharmacology, College of Pharmacy, University of Minnesota, Minneapolis, MN 55455, USA; jung0433@umn.edu

**Keywords:** Parkinson’s disease, amyotrophic lateral sclerosis, axonal transport, neuroinflammation, complement, *TREM2*, integrated stress response, GCN2, single-nucleus RNA sequencing, batch effect adjustment

## Abstract

Failure of axonal maintenance is proposed as a mechanism shared by Parkinson’s disease (PD) and amyotrophic lateral sclerosis (ALS). We reanalysed three public post-mortem resources under one rule set: bulk RNA-seq of 1242 samples from 319 donors (GSE153960) and midbrain single-nucleus RNA-seq (GSE157783, GSE178265). As a contributing project, a second batch variable tracked diagnosis in both cord segments and was completely separated from it in three cortical regions, which we removed. Adjusting for this removed a third of the naive differential expression. In ALS cord, the dominant depleted program was microtubule-based axonal transport (normalised enrichment score −2.40, FDR < 0.001); a regeneration-associated panel reached significance in none of ten fits. An ALS cord signature carried into the PD midbrain and scored highest on microglia in all 11 donors (+3.55 versus +0.73 next). Of 86 gene sets significant in both diseases, a translation block contained the GCN2 amino-acid-deficiency response. Both axes are compartment-level: within PD microglia, the complement panel is null (+0.21, *p* = 0.57). No GCN2 activity was measured, and the ALS cord is compared against PD midbrain. The axes that survive this control are glial and translational; the axonal question is not adjudicable in PD, where the panel score tracks dopaminergic content.

## 1. Introduction

Parkinson’s disease (PD) and amyotrophic lateral sclerosis (ALS) destroy different neurons in different places, yet both target cells carrying an axonal compartment far larger than the cell body that supplies it. Single-axon reconstruction in the rat shows that one nigrostriatal dopaminergic neuron sustains a vast, largely unmyelinated striatal arborisation [1,2], and the cost of maintaining and repeatedly depolarising it is a leading explanation for the selective loss of these neurons in PD [3,4]. Spinal motor neurons carry the same burden in the opposite geometry: a single axon that can span the length of a limb, with a neuromuscular terminal field at its far end. ALS begins there, with distal axonal and synaptic loss preceding death of the motor neuron cell body in mice and in human tissue [5,6]; the causative genes include the kinesin heavy chain *KIF5A* [7,8] and the dynactin subunit *DCTN1* [9,10]; and disrupting dynein–dynactin in mice produces progressive motor neuron degeneration [11]. One hypothesis follows from the two literatures together: that failure to maintain a long axon is a mechanism common to both diseases.

This hypothesis is asserted more often than it is measured. Its strongest support comes from systems in which a single gene is mutated, and a single compartment imaged [12,13], where transport deficits and axonal degeneration can in any case progress independently of one another [14]. Human evidence is harder to assemble, because the two diseases are studied in different tissue and at different resolution—ALS in spinal cord, largely in bulk RNA-seq; PD in midbrain, increasingly in single-nucleus data—so any claim of sharing sets one kind of measurement against another. Post-mortem consortium resources add a second difficulty. They are built from several contributing tissue banks over a period of years, and contributing site and library chemistry can track diagnosis; a batch variable correlated with diagnosis produces differential expression that downstream annotation cannot distinguish from a disease effect. Degeneration also changes the cellular composition of the tissue it affects, so a change measured in tissue is not by itself a change in expression per cell. Glial activation in the ALS spinal cord has already been described from this resource [15]; what has not been asked is where that signature falls when it is carried into a second disease as an externally defined variable. These are the properties a cross-disease comparison must handle, and they define the gap this study addresses.

We therefore reanalysed three public human post-mortem datasets under one set of rules rather than comparing published gene lists. GSE153960 supplies the ALS arm, and bulk RNA-seq was contributed by the New York Genome Center ALS Consortium and the Target ALS Human Post-mortem Tissue Core [15,16], of which 1242 samples from 319 donors enter the primary comparison. GSE157783 supplies annotated midbrain single-nucleus RNA-seq [17], and GSE178265 supplies substantia nigra single-nucleus RNA-seq with fluorescence-activated sorting of dopaminergic libraries [18], whose deposited diagnosis field records only disease versus control and therefore does not separate Parkinson’s disease from dementia with Lewy bodies. We modelled the batch variables of the ALS resource and removed the regions in which the contributing project is completely separated from diagnosis, because no design can identify a disease effect there. We aggregated nuclei to donor pseudobulk before any test, taking the donor rather than the nucleus as the unit of replication [19], and we applied one expression filter and one significance rule to every comparison. Curated panel statistics were refitted in the second cord segment and in each contributing cohort alone, and the number of fits in which each holds is reported with it rather than used as a filter; only two of those ten fits carry a composition covariate, and membership sensitivity was tested on one panel (Section 4).

What emerges does not follow the shape of the hypothesis. In the ALS spinal cord, the dominant depleted program is microtubule-based axonal transport (NES = −2.40, FDR < 0.001 in cervical cord), and the lumbar cord names retrograde and anterograde axonal transport directly. A ten-gene axonal structure-and-transport panel containing *KIF5A* and *DCTN1* is depleted in nine of the ten ALS analyses fitted, across cord segment, contributing cohort, and covariate model, and both genes carry negative estimates in both segments. Whether that deficit transfers cannot be determined here. In dopaminergic-enriched nigral nuclei, the transport-motor panel is positive rather than depleted (median Wald +1.41). But the donor-level panel score falls as that donor’s dopaminergic content rises (ρ = −0.525, *p* = 0.031), and disease donors carry less of that content, so this contrast settles the question of an ALS-like transport deficit in PD in neither direction. Where the two diseases meet is glial and translational: a complement and phagocytosis program, and a shared block of translation and ribosome gene sets that includes the Reactome response of *EIF2AK4* (GCN2) to amino acid deficiency [20]. Neither is a per-cell claim. The complement result is a compartment-level observation: the same panel is flat within PD microglial pseudobulk, and in bulk cord, a glial rise can follow from neuronal loss alone. An ALS-derived cord signature carried into the PD midbrain ranks microglia first in all 11 donors, which states where those transcripts sit rather than that a diagnosis raises them. The shared translation block is named by annotation rather than by any measurement of kinase activity, and it is a rank-level property of gene sets that does not localise to the dopaminergic compartment. A regeneration-associated gene panel reaches significance in none of the ALS models fitted. Because the ALS contrast is cord and the PD contrast is midbrain, what follows describes where the two diseases place a common axis, not whether that axis is theirs alone.

## 2. Results

### 2.1. Contributing Project Is Confounded with Diagnosis, and in Three Regions the Confounding Is Complete

**Figure 1:**
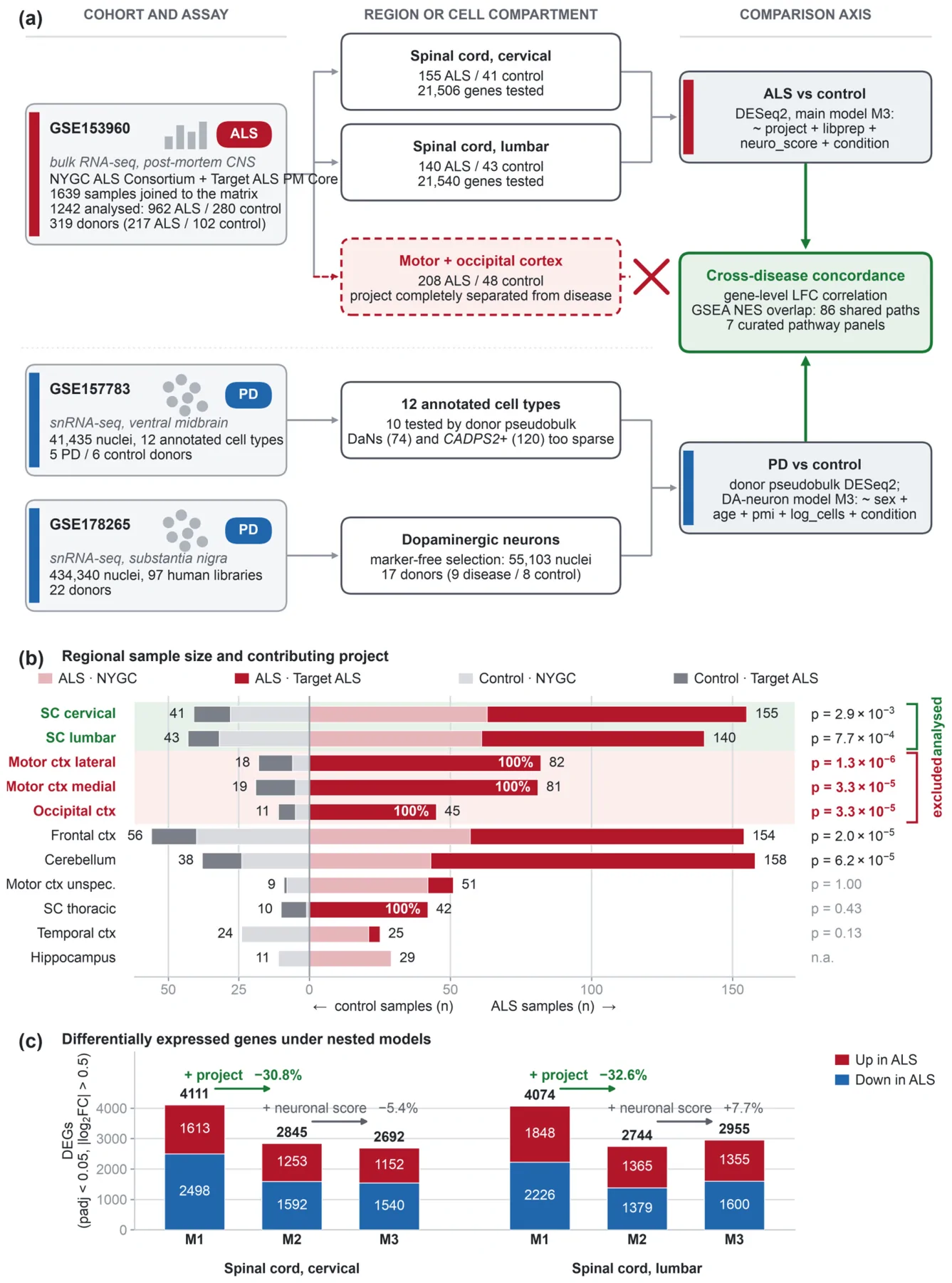
Study design, the batch structure of the ALS cohort, and differential-expression counts under nested covariate models. (**a**) Analysis scheme: three public datasets (left) enter two within-disease contrasts (right) meeting in one cross-disease comparison (green box). GSE153960 [15,16] contributes 1242 analysed samples (962 ALS, 280 control) from 319 donors; GSE157783 [17] 41,435 nuclei in 12 cell types from 5 PD and 6 control donors; GSE178265 [18] 55,103 selected nuclei from 18 donors, 17 of which (9 disease, 8 control) enter the contrast. Disease is the deposit’s own diagnosis label and does not resolve further. Design formulas are printed in the contrast boxes. The red dashed box and ✕ mark the motor and occipital cortex samples (208 ALS, 48 control) excluded from the cross-disease comparison. (**b**) Sample size and contributing project per region. Bars extend left for controls, right for ALS; group sizes at the bar ends. Light pink, ALS·NYGC; dark red, ALS·Target ALS; light grey, control·NYGC; dark grey, control·Target ALS; in-bar “100%”, regions whose ALS samples all come from Target ALS. *p*, two-sided χ^2^ of project against diagnosis within region, uncorrected; n.a. where undefined. Green shading and bracket, regions carried forward; red, excluded. (**c**) Genes at *p*_adj_ < 0.05 and |log_2_FC| > 0.5 in cervical (left) and lumbar (right) cord under M1, ~libprep + condition; M2, ~project + libprep + condition; M3, ~project + libprep + neuro_score + condition. Bars stack by direction (red, higher in ALS; blue, lower), counts inside and totals above; green arrows, percentage change on adding project; grey arrows, on adding neuronal content.

The ALS arm rests on 1242 bulk RNA-seq samples from 319 donors, recovered from a 1659-column matrix by exact metadata matching. ALS is represented by GSE153960, a post-mortem bulk RNA-seq collection of the New York Genome Center (NYGC) ALS Consortium and the Target ALS Human Post-mortem Tissue Core [15,16]. Its matrix holds 1659 samples across 58,929 genes. Exact matching on the sample ID alt key recovered a GEO record for 1639 of them. One further sample was removed because a single key mapped to two libraries. Restricting to the two unambiguous diagnostic groups left 1242 samples: 962 from ALS-spectrum motor neuron disease and 280 from non-neurological controls, contributed by 217 and 102 donors (Figure 1a; Table S1). Donors gave a median of four samples each, maximum nine, but at most one sample per region outside the cerebellum, so every region-wise analysis below runs on independent donors (Figure S1c,d).

The PD side rests on two midbrain single-nucleus resources of different designs. GSE157783 [17] contributes 41,435 nuclei annotated by the original authors into 12 cell types, from five PD and six control donors as recorded in the GEO deposit. GSE178265 [18] contributes a publicly processed matrix of 434,340 nuclei from 97 human libraries and 22 donors; its deposited diagnosis field takes only the values Ctrl and Disease, so Parkinson’s disease cannot be separated from dementia with Lewy bodies within it (Figure 1a).

Two technical variables track diagnosis in the ALS resource, and they are not on the same axis. Library preparation chemistry is distributed unevenly between groups overall (χ^2^ *p* = 6.1 × 10^−5^, *n* = 1242 samples). Within the two cord segments that carry the analysis, it is balanced (cervical χ^2^ *p* = 0.859, *n* = 196; lumbar χ^2^ *p* = 0.564, *n* = 183; Figure S1a). Contributing project is the stronger variable: pooled across regions, it is associated with diagnosis at χ^2^ *p* = 1.12 × 10^−22^ (*n* = 1242 samples). The imbalance persists inside the cervical cord, where 59.4% of ALS samples but 31.7% of controls come from Target ALS (92/155 versus 13/41; χ^2^ *p* = 2.88 × 10^−3^), and inside the lumbar cord (56.4% versus 25.6%; 79/140 versus 11/43; χ^2^ *p* = 7.67 × 10^−4^) (Figure 1b). Project and chemistry are themselves associated (χ^2^ *p* = 3.87 × 10^−69^; the NYGC contribution is 87% Automated, 437/500; the Target ALS contribution is 63% Manual, 468/742). Adjusting for chemistry alone therefore leaves the project effect in place (Figure S1b).

In the lateral motor cortex, medial motor cortex, and occipital cortex, the association is a complete separation rather than an imbalance. Every ALS sample in those three regions comes from Target ALS—82/82, 81/81, and 45/45—while controls are drawn from both projects (66.7%, 73.7%, and 54.5% Target ALS; χ^2^ *p* = 1.27 × 10^−6^, 3.30 × 10^−5^, and 3.33 × 10^−5^) (Figure 1b). No model separates a disease effect from a project effect when one arm of the contrast has a single source. We therefore removed these three regions, and with them, 208 ALS and 48 control samples, from differential expression rather than reporting estimates that are not identifiable.

Adjustment for project removes about a third of the naive differential expression. Cervical cord fitted with chemistry alone returns 4111 genes at adjusted *p* < 0.05 and |log_2_ fold change| > 0.5; adding project returns 2845, a fall of 30.8%. Lumbar cord falls from 4074 to 2744, a fall of 32.6% (Figure 1c). Adding a pan-neuronal composition covariate gives the final model: ~project + libprep + neuro_score + condition. This model returns 2692 genes in cervical cord (155 ALS, 41 control; 21,506 genes tested) and 2955 in lumbar cord (140 ALS, 43 control; 21,540 tested) (Table S4). Of the 2692 cervical calls, 2498 were already significant under both simpler designs, so the adjustments overwhelmingly subtract genes: only 58 of the 2692 are significant under neither simpler design (Figure S3d).

Modelling neuronal content removes the pan-neuronal signal but leaves motor-neuron-restricted loss in place. Neuronal content differs between groups in direction but not significantly: the twelve-gene pan-neuronal proxy z-score is −0.083 in ALS and +0.313 in controls (Mann–Whitney *p* = 0.087; *n* = 196 cervical samples; Figure S3b; Table S2). The twelve-gene pan-neuronal panel moves from a median Wald statistic of −2.19 against a protein-coding background before adjustment (*p* = 0.021) to −2.25 after it (*p* = 0.278), so the panel no longer separates from that background once neuronal content is modelled (Figure S3c). Motor-neuron-restricted markers survive the adjustment: *MNX1* falls by 1.63 log_2_ units before it (adjusted *p* = 1.0 × 10^−6^) and by 1.47 after, and *SLC18A3* by 1.12 (adjusted *p* = 9.4 × 10^−4^) and 0.98. The generic neuronal markers behave differently. *RBFOX3* falls by 0.63 before adjustment (adjusted *p* = 0.044) and by 0.20 after, at which point it no longer reaches significance (adjusted *p* = 0.125). *SNAP25* moves the other way: flat before adjustment (log_2_ fold change −0.08, adjusted *p* = 0.79); it rises after adjustment (+0.57, adjusted *p* = 8.3 × 10^−10^) (Figure S3a).

### 2.2. The ALS Cord Signature Reproduces Across a Second Cord Segment and Across a Second Contributing Cohort

**Figure 2:**
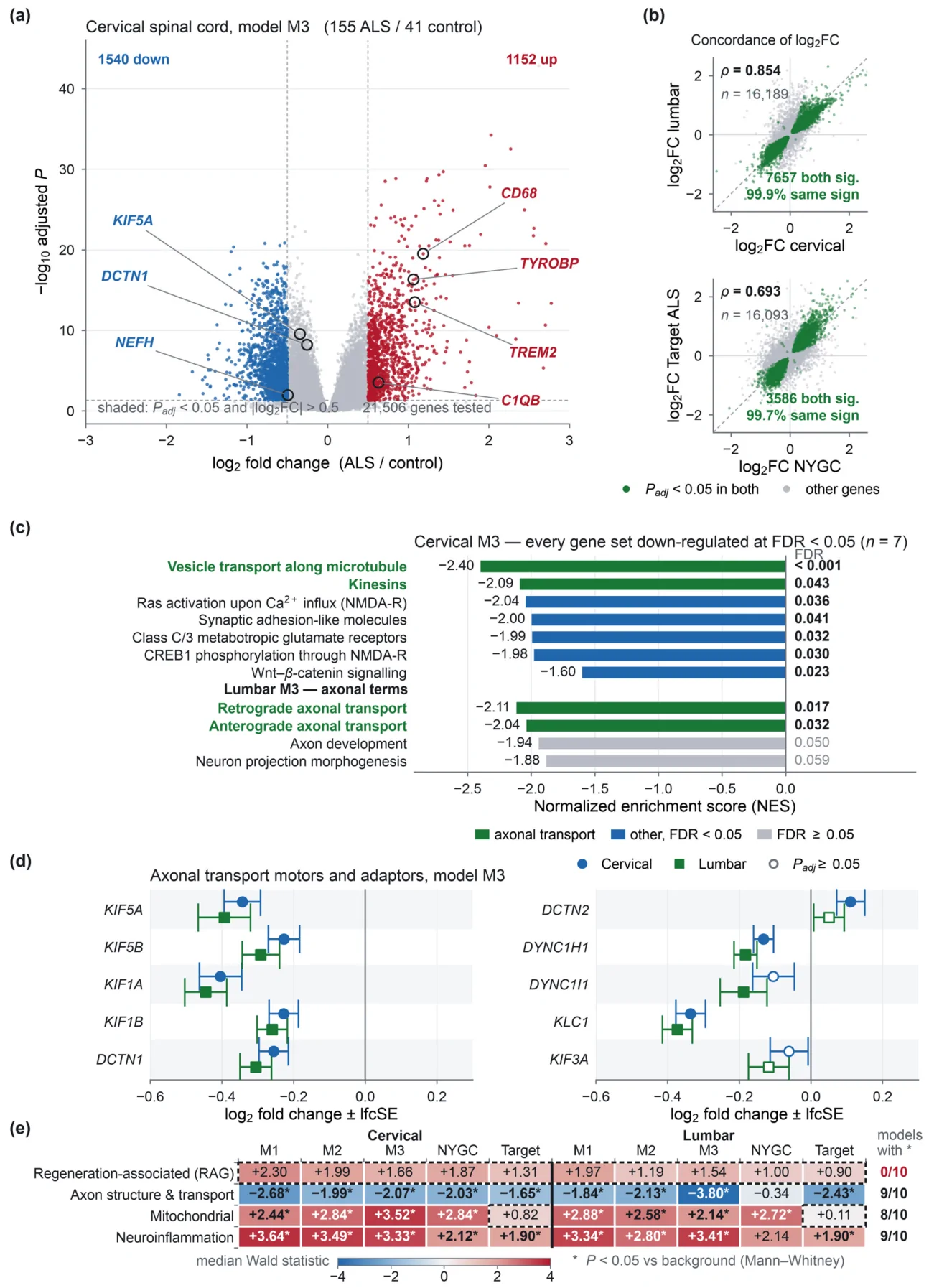
Differential expression in the ALS spinal cord, its reproduction across cord segment and contributing cohort, and four curated gene panels across ten analyses. All values are from GSE153960 under M3 (~project + libprep + neuro_score + condition), fitted per segment with PyDESeq2 (Wald test, Benjamini–Hochberg adjustment), unless stated. (**a**) Volcano plot of cervical cord (155 ALS, 41 control; 21,506 genes tested). Points are coloured only when they pass both thresholds—red, higher in ALS; blue, lower; grey otherwise; dashed verticals mark log_2_FC = ±0.5, and the dashed horizontal line marks *p*_adj_ = 0.05. Counts appear at the top of each half. Black rings mark the seven named genes, whose labels are coloured by the sign of the fold change and not by significance; *KIF5A*, *DCTN1*, and *NEFH* fall inside the fold-change band, and their points are therefore grey. (**b**) Gene-wise log_2_ fold changes across cord segment (top, cervical versus lumbar under M3) and contributing cohort (bottom, NYGC versus Target ALS in cervical cord, each stratum fitted with ~libprep + condition). Green, genes at *p*_adj_ < 0.05 in both; grey, others; dashed line, identity. ρ is Spearman over genes quantified in both (*n* in panel); the green annotation gives the doubly significant count and the percentage sharing sign. (**c**) Normalised enrichment scores (NES) from preranked gene-set enrichment analysis, genes ranked by Wald statistic. Upper block, every set at FDR < 0.05 with negative NES in cervical cord (*n* = 7); lower block, the four most depleted of 27 axonal terms in lumbar cord. Green, axonal-transport terms; blue, other terms at FDR < 0.05; grey, FDR ≥ 0.05; FDR printed at right. (**d**) log_2_ fold change ± lfcSE for the ten axonal transport motor and adaptor genes. Blue circles, cervical; green squares, lumbar; open symbols, *p*_adj_ ≥ 0.05. (**e**) Median Wald statistic of four panels across five analyses per segment (the three nested models and the two cohort strata). Panel sizes 10, 10, 17, and 16 genes; the neuroinflammation panel is realised on 15 of its 16 in the cervical Target ALS stratum. Colour, median on the −4 to +4 scale below; asterisk, two-sided Mann–Whitney *p* < 0.05 against all protein-coding genes tested in the same comparison, uncorrected. The right column counts starred cells of ten and is set in red where that count is zero; the dashed outlines are annotation only and enclose the regeneration-associated row and the two Target ALS cells of the mitochondrial row.

The cervical cord contrast splits along a single axis: axonal transport and neurofilament transcripts fall; microglial complement and phagocytosis transcripts rise. Under the final model, it returns 1152 up-regulated and 1540 down-regulated genes of 21,506 tested (155 ALS and 41 control). *KIF5A* (−0.34), *DCTN1* (−0.26), and *NEFH* (−0.49) all carry negative estimates at adjusted *p* < 0.05, but their fold changes fall inside the 0.5 band, and they are therefore not counted among the 1540. *CD68*, *TYROBP*, *TREM2*, and *C1QB* clear both thresholds on the up side (Figure 2a). Glial activation in the ALS spinal cord is already reported from this consortium resource [15]; what the design below adds is where that signature falls when it is carried into a second disease as an externally defined variable. The lumbar cord splits comparably: 1355 up and 1600 down of 21,540 tested.

The same effect sizes reappear at a second anatomical level of the cord. Across the 16,189 genes quantified in both segments, the gene-wise log_2_ fold changes correlate at Spearman ρ = 0.854. Of the 7657 genes significant in both, 99.9% move in the same direction (Figure 2b).

They also reappear when each project is analysed on its own. The cervical strata hold 63 ALS versus 28 controls for NYGC and 92 versus 13 for Target ALS. Refitted separately, the two cohorts agree at ρ = 0.693 in cervical cord (*n* = 16,093 genes; 3586 significant in both, 99.7% concordant) (Figure 2b), and at ρ = 0.695 in lumbar cord (*n* = 16,148; 3125 significant in both, 99.7% concordant). At the gene-set level, 588 pathways reach FDR < 0.05 in the NYGC stratum and 511 in the Target ALS stratum, of which 326 unique terms are shared (Table S5). Enrichment scores of the two strata correlate at ρ = 0.767 over the 3909 terms tested in both, and all 326 shared terms agree in sign. The stratified fits are thus a weaker but fully independent estimate of every effect below.

### 2.3. Microtubule-Based Axonal Transport Is the Dominant Depleted Program, and No Model Supports Activation of a Regeneration Program

Only seven gene sets are depleted at FDR < 0.05 in the cervical cord, and the two strongest are transport machinery. Vesicle transport along microtubules reaches a normalised enrichment score (NES) of −2.40 (FDR < 0.001), and Kinesins NES = −2.09 (FDR = 0.043). The remaining five are synaptic and receptor programs: Ras activation upon Ca^2+^ influx through the NMDA receptor (−2.04), synaptic adhesion-like molecules (−2.00), class C/3 metabotropic glutamate receptors (−1.99), *CREB1* phosphorylation through NMDA receptor activation (−1.98), and Wnt–β-catenin signalling (−1.60) (Figure 2c). This depletion sits against 570 significant gene sets in total for the region, so it is a narrow and specific minority.

Lumbar cord names the same machinery explicitly. Retrograde axonal transport reaches NES = −2.11 (FDR = 0.017) and anterograde axonal transport NES = −2.04 (FDR = 0.032). Axon development (−1.94, FDR = 0.050) and neuron projection morphogenesis (−1.88, FDR = 0.059) follow close behind (Figure 2c). The direction is coherent across the whole annotation, not only in the significant tail. Of the 28 axon-related gene sets tested—a list purged of axonemal, that is, ciliary, terms, which share the string but not the biology—22 carry a negative enrichment score in cervical cord, at a median NES of −1.34. Lumbar cord gives 22 of 27.

At the gene level, nine of the ten transport motors and adaptors examined carry a negative estimate in both segments, and seven of those nine are significant in both. *KIF5A* is an established ALS gene [7,8]; its log_2_ fold change is −0.34 ± 0.05 in cervical and −0.39 ± 0.07 in lumbar cord (adjusted *p* = 2.9 × 10^−10^ and 6.0 × 10^−7^). The dynactin subunit *DCTN1*, mutated in motor neuron disease [9,10], changes by −0.26 ± 0.04 and −0.31 ± 0.04. *KIF1A* (−0.40 ± 0.06 and −0.45 ± 0.06), *KLC1* (−0.34 ± 0.04 and −0.37 ± 0.04), *KIF5B*, *KIF1B*, and *DYNC1H1* follow. The single exception is the dynactin subunit *DCTN2*, which rises modestly in the cervical cord (+0.11 ± 0.04, adjusted *p* = 0.011) and does not move in the lumbar cord (+0.05 ± 0.04, adjusted *p* = 0.31) (Figure 2d). The neurofilament *NEFH*, which is not a member of this panel, changes by −0.49 in cervical and −0.75 in lumbar cord (adjusted *p* < 0.05 in both; Figure 2a).

The transport deficit survives every change in model and cohort but one. We fitted ten analyses: three nested designs and two cohort strata, in each of two cord segments. The ten-gene axonal structure-and-transport panel is significant against a protein-coding background in nine of them, with a median Wald statistic of −2.07 under the final cervical model (*p* = 0.026). It fails only in the NYGC lumbar stratum (−0.34, *p* = 0.134). Neuroinflammation is significant in nine of ten fits. The 17-gene mitochondrial panel is significant in eight, failing in both Target ALS strata (+0.82, *p* = 0.34 cervical; +0.11, *p* = 0.97 lumbar), which places oxidative phosphorylation on weaker ground than transport (Figure 2e).

The regeneration-associated panel is significant in none of the ten fits. Its median Wald statistic is +1.66 in cervical cord under the final model (*p* = 0.108, *n* = 10 genes) and +1.54 in lumbar cord (*p* = 0.501). The smallest *p* value anywhere in the ten fits is 0.050, in the NYGC cervical stratum, where the panel median is +1.87 (*n* = 10 genes; Figure 2e). Individual members do rise: *GAP43* (Wald +3.98, adjusted *p* = 2.7 × 10^−4^), *KLF6* (+3.21, *p* = 0.0037), *SMAD1* (+2.77, *p* = 0.013), and *GADD45A* (+2.68, *p* = 0.016), while *KLF7* moves against them (−2.80, *p* = 0.012). Panel-level significance depends on which genes are counted. A nine-gene version that omits *KLF7* reaches *p* = 0.044 in the cervical cord, and dropping any one of *KLF6*, *GAP43*, *GADD45A*, *SMAD1*, or *CREB1* from that version returns it above threshold (*p* = 0.076, 0.088, 0.067, 0.069, and 0.056; Figure S3e). The increases are therefore confined to individual genes, and no fit gives panel-level evidence of regeneration-program activation. This leave-one-out analysis was run on the regeneration panel alone; the transport and complement conclusions do not rest on their panel statistics but on the gene-set enrichments and single-gene effect sizes reported with them.

### 2.4. The Axis Shared by the Two Diseases Is a Microglial Complement and Phagocytosis Program, Not a Neuronal One

**Figure 3:**
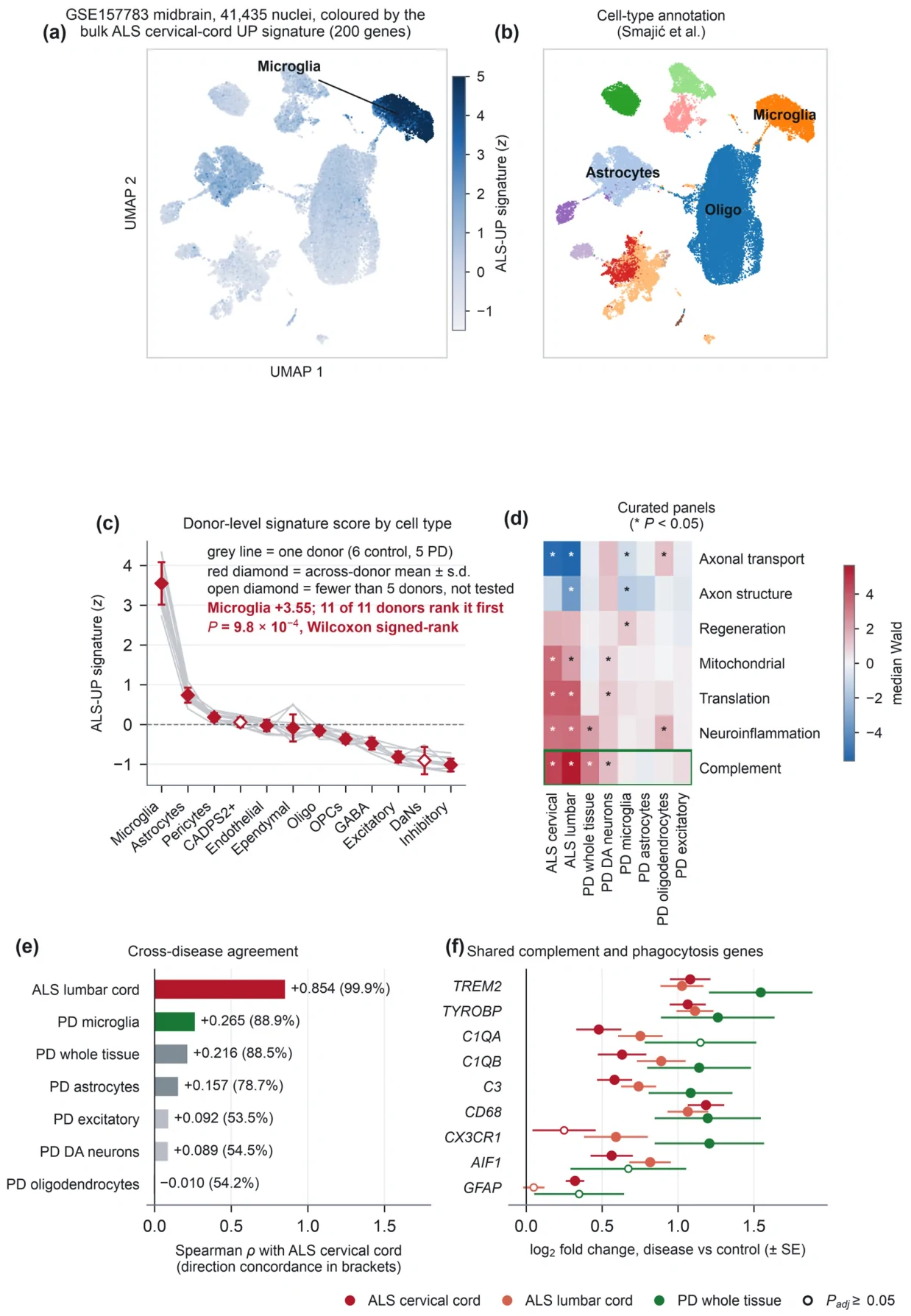
Localisation of an ALS-derived cord signature in the PD midbrain, curated pathway panels across eight compartments, gene-level concordance with ALS cervical cord, and the shared complement and phagocytosis genes. ALS cervical and lumbar cord are GSE153960 under M3. PD whole tissue, microglia, astrocytes, oligodendrocytes, and excitatory neurons are donor pseudobulk of GSE157783 [17] (5 PD, 6 control). Adjusted *p* values are Benjamini–Hochberg. PD dopaminergic neurons are pseudobulk of the GSE178265 selection [18] (9 disease, 8 control), whose deposited diagnosis field distinguishes only Ctrl from Disease. (**a**) The 41,435 GSE157783 nuclei in the embedding of Figure S5a, coloured by the score_genes score of the 200 genes most up-regulated in ALS cervical cord. The colouring variable is defined in GSE153960, not in these data. Colour bar, per-nucleus score (*z*), clipped to [−1.5, +5]; a leader line labels the microglial cluster; axes are UMAP coordinates without units. (**b**) The same nuclei coloured by the authors’ cell-type annotation [17]; all twelve classes are named on the axis of panel (**c**). (**c**) The same score as donor means, one grey line per donor (6 control, 5 PD), cell types ordered by across-donor mean. Red-filled diamonds, mean ± s.d.; open diamonds, types reaching the nucleus threshold in fewer than 5 donors, not tested; dashed line, zero. *p* is a two-sided Wilcoxon signed-rank test over 11 donors, uncorrected; 9.8 × 10^−4^ is its smallest attainable value at *n* = 11. (**d**) Median Wald statistic of seven panels in eight compartments (columns left to right: ALS cervical, ALS lumbar, PD whole tissue, PD dopaminergic, PD microglia, PD astrocytes, PD oligodendrocytes, and PD excitatory). Colour, median statistic on a scale running to the largest absolute median in the panel (colour bar at right); asterisk, two-sided Mann–Whitney *p* < 0.05, uncorrected; green outline, the complement and phagocytosis row. Realised membership differs between columns, so cells are compared within a column and only by direction across columns (Section 4.8; Table S2). (**e**) Spearman correlation of gene-wise log_2_ fold change with ALS cervical cord; the bracketed percentage is the share of doubly significant genes agreeing in direction. Red, ALS lumbar cord; green, PD microglia; grey elsewhere, darker where ρ ≥ 0.12, a display threshold and not a test. Genes quantified in both range from 10,205 (microglia) to 16,189 (ALS lumbar). (**f**) log_2_ fold change ± 1 SE for nine complement, phagocytosis and glial-activation genes. Red, ALS cervical; salmon, ALS lumbar; green, PD whole tissue; filled, *p*_adj_ < 0.05; open, *p*_adj_ ≥ 0.05; vertical line, zero.

The gene signature raised in the ALS spinal cord lands on one compartment of the PD midbrain. Scoring all 41,435 GSE157783 nuclei on the 200 genes most strongly raised in ALS cervical cord places the highest values on microglia, at a median nucleus-level *z* of +3.59 against +0.69 for astrocytes, the next-ranked class (Figure 3a,b). Aggregated to donor means, microglia reach +3.55 (s.d. 0.536 across 11 donors) against +0.73 for astrocytes, while the tested neuronal compartments are negative, at −0.82 for excitatory and −1.02 for inhibitory neurons. All 11 donors rank microglia first, at donor scores of +2.73 to +4.33 (each donor’s microglial mean against that same donor’s mean over all testable cell types, two-sided Wilcoxon signed-rank *p* = 9.8 × 10^−4^ unadjusted, *n* = 11 donors; Figure 3c), which is the smallest two-sided value the test can return at this number of donors. Dopaminergic and *CADPS2*-positive neurons reach the nucleus threshold in fewer than five donors and were not tested. The scoring variable is defined in the ALS bulk data and enters the PD atlas from outside it, so this placement is not a restatement of the published midbrain annotation. That the placement is microglial is itself a property of the signature rather than of the test: of the 190 up-signature genes present in GSE157783, 111 (58.4%) reach their highest mean expression in microglia, against 10.5% of all expressed genes and a mean of 19.7 genes (10.3%, 95% range 12–28) across 10,000 gene sets matched to the signature on mean expression (empirical *p* < 1 × 10^−4^; 5.65-fold). Removing all 111 leaves 79 genes on which astrocytes rank first (+2.29) and microglia fourth (+0.79), so the microglia-first ordering rests on those genes. On the reduced score, the five highest compartments are all non-neuronal—astrocytes +2.29, pericytes +1.09, ependymal +0.90, microglia +0.79, and endothelial cells +0.47—and no neuronal compartment is positive, although oligodendrocytes (−0.28) and oligodendrocyte precursors (−0.36) are negative as well (Section 4.5).

This placement indicates which cells carry the transcripts, not that expression per cell is raised. Within PD microglial pseudobulk itself, the complement and phagocytosis panel is null: median Wald +0.21, *p* = 0.57, on 9 of its 10 members, from 3903 microglial nuclei across 5 PD and 6 control donors (Figure 3d). A compartment-level increase with a null within-microglia test fits a larger or differently distributed microglial population as readily as higher expression per microglial cell, and these data do not separate the two. The same panel scores +1.38 in dopaminergic-enriched nuclei (*p* = 6.1 × 10^−3^, 9 of 10 members realised), a compartment in which these transcripts are not expected to originate.

Two of seven curated panels change significantly in the same direction in ALS spinal cord and in PD whole midbrain tissue, and both are glial (Figure 3d). Complement and phagocytosis reach a median Wald statistic of +4.44 in ALS cervical cord (*p* = 3.8 × 10^−4^), +5.53 in ALS lumbar cord (*p* = 5.3 × 10^−5^), and +3.12 in PD whole midbrain tissue (*p* = 2.2 × 10^−6^), on 10, 10, and 9 of its 10 members. Neuroinflammation reaches +3.33, +3.41, and +2.15 in the same three compartments (*p* = 0.016, 0.035, and 2.9 × 10^−7^), in 16, 16, and 13 of its 16 members. Axonal transport does not behave this way: it is strongly negative only in the ALS cord (−5.38 and −5.64) and is positive in the dopaminergic-enriched PD compartment (+1.41, *p* = 0.082) (Figure 3d). These 56 panel tests are uncorrected; each panel is a subset of the background it is tested against, and values realised on different memberships are not comparable across datasets (Section 4.8). The statistic therefore orders panels rather than establishing any one of them, and the shared axis rests on the gene-level and gene-set results that follow.

Compartment-wise concordance places the shared signal in microglia. Gene-wise effect sizes were compared against ALS cervical cord for each PD compartment. Agreement is highest for microglia (ρ = 0.265 over 10,205 shared genes; 88.9% directional concordance among the 279 genes significant in both) and for whole midbrain tissue (ρ = 0.216 over 14,681; 88.5% of 269). Astrocytes are lower (ρ = 0.157 over 11,853; 78.7% of 136). Neither neuronal compartment separates from a coin flip in direction, and neither does the oligodendrocyte comparison: excitatory neurons ρ = 0.092 (13,166 genes; 53.5% of 99 doubly significant genes concordant), dopaminergic neurons ρ = 0.089 (14,613; 54.5% of 235), and oligodendrocytes ρ = −0.010 (13,130; 54.2% of 240) (Figure 3e). Oligodendrocytes sit at the bottom of this ordering despite contributing 507 differentially expressed genes, more than any other PD cell type, so their position is not a matter of detection power. The coefficients themselves are small and closely spaced; whole tissue contains microglia, and the compartments differ in the genes and donors available, so this ordering alone does not separate microglia from glia in general; the donor-level test above does.

PD microglia occupy a specific inflammatory state rather than a generic activated one. Their pseudobulk contrast (5 PD, 6 control; 11,215 genes tested) returns 418 differentially expressed genes, 54 up and 364 down, and 84 significant gene sets, 23 of them enriched and 61 depleted (Figure S4a,b; Table S6). Among the enriched terms are HSF1 activation (NES = +2.23, FDR = 0.009), NIK/NF-κB signalling (+2.16, FDR = 0.025), and NLR-family inflammasome components (+2.12, FDR = 0.019). The most depleted are nervous system development (−2.33), central nervous system development (−2.26), axon development (−2.26), and protein–protein interactions at synapses (−2.24), all at FDR < 0.001 (Figure S6a; Table S6).

PD whole midbrain tissue is dominated by immune annotation, with one qualification attached to the annotation itself. Of its 472 significant gene sets, the four strongest are immune terms: regulation of immune response (NES = +2.66), immunoregulatory interactions between lymphoid and non-lymphoid cells (+2.63), regulation of interleukin-6 production (+2.60), and defence response to bacterium (+2.59). Of those 472 sets, 434 are enriched and 38 depleted, and the depleted tail is shallow: the most negative are HCMV late events (−2.11, FDR = 0.018), defective TPR in thyroid papillary carcinoma (−1.93, FDR = 0.049), and NoRC-mediated rRNA repression (−1.90, FDR = 0.049); all three are plotted in Figure S4c. GSE157783 carries no cytoplasmic ribosomal protein genes at all, and only 6 of the 14 members of our translation panel exist in it, compared with 14 of 14 in GSE178265 (Table S2). Translation gene sets built from that annotation are correspondingly shrunken or fall below the minimum-size filter, so the immune dominance of the whole-tissue ranking is in part a property of the deposited matrix.

Five genes carry the shared axis in all three bulk compartments—ALS cervical cord (155 ALS and 41 control), ALS lumbar cord (140 ALS and 43 control), and PD whole midbrain tissue (5 PD and 6 control). *TREM2* rises by log_2_ fold change +1.08, +1.03, and +1.55 in ALS cervical cord, ALS lumbar cord, and PD whole tissue. *TYROBP* rises by +1.06, +1.11, and +1.26; *C1QB* by +0.63, +0.89, and +1.14; *C3* by +0.58, +0.74, and +1.08; and *CD68* by +1.18, +1.06, and +1.20. All five are significant in all three compartments. *C1QA*, *CX3CR1*, *AIF1* and *GFAP* are significant in some compartments and not others (Figure 3f). In the 12 annotated cell types of GSE157783, eight of these nine genes reach their highest mean expression in microglia, *GFAP* being the astrocyte-dominant exception (Figure S6b).

Two controls that would bound this comparison do not exist in these resources. The ALS contrast is spinal cord, and the PD contrast is midbrain, so anatomical region and diagnosis cannot be separated here. The other neurological-disease samples of GSE153960 are completely separated from the comparison groups in the contributing project (Section 4.3; Figure S2c), so no alternative-diagnosis control is available either. A microglial complement and phagocytosis response is a common feature of degenerating central nervous system tissue [21,22,23], and these data place the shared axis in microglia without establishing that it is specific to these two diseases.

### 2.5. The Two Diseases Share the GCN2 Arm of the Integrated Stress Response, and It Does Not Sit in the Dopaminergic Compartment

The dopaminergic contrast needed a compartment the annotated midbrain atlas cannot supply, since that atlas holds 74 dopaminergic nuclei in total. We therefore selected dopaminergic-enriched nuclei from GSE178265 by a rule that uses neither dopaminergic nor axonal genes. The deposited diagnosis field of that resource records only Ctrl and Disease, so its disease donors form a nigral degeneration group in which Parkinson’s disease is not separated from dementia with Lewy bodies, and every statement below about PD dopaminergic neurons is bounded by that. The rule combines origin in a substantia nigra library sorted as *NR4A2*-positive by fluorescence-activated cell sorting (FACS) with a floor on the pan-neuronal marker score and ceilings on the oligodendrocyte, astrocyte, and microglial scores, all expressed per 10,000 unique molecular identifiers (UMI) (Section 4.6). It yields 55,103 nuclei from 18 donors, 17 of which pass a floor of 100 nuclei (9 disease, 8 control; 27,415 of 41,625 genes tested; Table S3). The compartment is dopaminergic-enriched rather than dopaminergic-pure: of the 55,103 nuclei, 12,973 (23.5%) fall in the eleven clusters in which at least half the nuclei reach a dopaminergic marker score of 5. Every donor-level contrast below is fitted on the whole selection, which the figures label as PD dopaminergic neurons.

Three checks bound that selection (Figure S7a–c). The caudate nucleus receives dopaminergic projections but holds no dopaminergic somata, and it contributes 0.005% marker-high nuclei—those whose dopaminergic marker score exceeds 5 per 10,000 UMI—against 6.211% in substantia nigra (2 of 40,467 versus 24,463 of 393,873). Nuclei carrying a dopaminergic marker score of at least 10 together with glial scores of at most 2 come 99.3% from FACS-positive libraries, against a 37.8% background across all nigral nuclei (Figure S7b). The selected population is neuron-rich and nearly free of oligodendrocyte signal, at median pan-neuronal and oligodendrocyte scores of 52.66 and 0.59 per 10,000 UMI against 2.42 and 12.70 in the 382,375 nuclei outside the marker-defined population, a group that overlaps the analysed selection rather than excluding it (Figure S7c). Dopaminergic content is lower in disease donors, at a marker-high fraction of 0.124 against 0.340 in controls (Mann–Whitney *p* = 0.027, *n* = 17 donors; Figures S5c and S7d). The dopaminergic identity panel falls with it (median Wald −1.54, *p* = 2.8 × 10^−4^, *n* = 9 genes; Figures S4d and S7e). In the unintegrated embedding, the selected compartment is self-organising: 12,973 nuclei in 11 Leiden clusters form a single connected component, at a k-nearest-neighbour purity of 0.951 against a chance expectation of 0.249 (Figure S5b).

**Figure 4:**
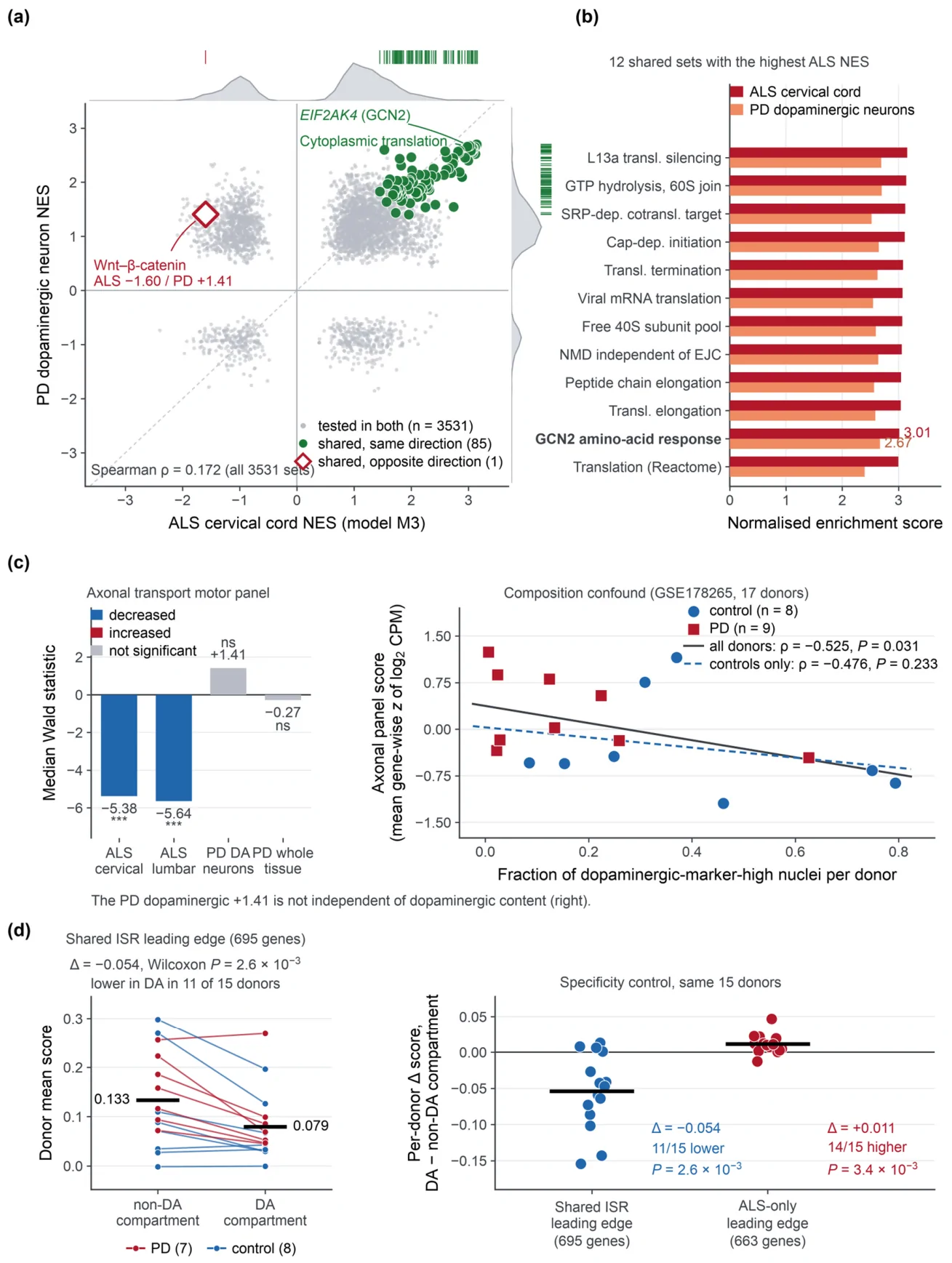
Gene-set overlap between ALS spinal cord and PD dopaminergic neurons, the axonal transport motor panel across compartments, and the compartment placement of the shared leading edge. (**a**) Normalised enrichment scores of the 3531 gene sets tested in both ALS cervical cord (M3, GSE153960) and PD dopaminergic neuron pseudobulk (GSE178265). Grey, all sets; green, the 85 at FDR < 0.05 in both with the same sign; open red diamond, the one significant in both with opposite signs (Wnt–β-catenin signalling, values beside it); dashed line, identity. Green labels name the Reactome *EIF2AK4* (GCN2) amino-acid-deficiency response and cytoplasmic translation. The marginal panels are histogram densities of the two NES distributions, with rug ticks marking the shared sets in green where the enrichment score is positive and red where it is negative. ρ is Spearman over all 3531 sets. (**b**) The 12 shared sets with the highest ALS NES as paired bars: dark red, ALS cervical cord; light orange, PD dopaminergic neurons, ordered by descending ALS NES; values printed for the GCN2 row only. (**c**) Left, median Wald statistic of the ten-gene axonal transport motor panel in four compartments. Blue, negative; red, positive; grey, not significant; two-sided Mann–Whitney against all protein-coding genes tested, uncorrected: *** *p* < 0.001, ns, not significant. Right, per-donor axonal panel score (mean gene-wise *z* of log_2_ CPM) against that donor’s marker-high fraction, for the 17 GSE178265 donors with ≥100 selected nuclei. Blue circles, control (*n* = 8); red squares, PD (*n* = 9); solid line, fit over all 17; dashed blue line, over the 8 controls; ρ and *p* are Spearman. A nucleus is marker-high when *da_s* > 5, the summed *TH*, *SLC6A3*, *SLC18A2*, and *DDC* count per 10,000 UMI. (**d**) Left, donor-paired comparison of the 695 leading-edge genes of the 86 shared sets, scored per nucleus across the 55,103 selected nuclei and averaged per donor. Each line joins one donor’s non-dopaminergic and dopaminergic compartments; red, PD (*n* = 7); blue, control (*n* = 8); the 15 donors plotted contribute ≥ 30 nuclei to both. Black bars, compartment means; Δ, the mean paired difference; *p*, two-sided Wilcoxon signed-rank over 15 donors, uncorrected. Right, the same difference for the shared leading edge (blue) and, as a specificity control, the ALS-only leading edge (red; 663 genes).

Of 3531 gene sets tested in both ALS cervical cord and PD dopaminergic neurons, 86 reach FDR < 0.05 in both, and 85 of those move in the same direction. The single exception is Wnt–β-catenin signalling, depleted in ALS (NES = −1.60, FDR = 0.023) and enriched in PD dopaminergic neurons (+1.41, FDR = 0.047), which we exclude from the shared block. Global agreement across all 3531 terms is weak (ρ = 0.172). The sharing is therefore concentrated in one block rather than transcriptome-wide (Figure 4a; Table S7).

The shared block is translation, and the GCN2 arm of the integrated stress response sits inside it. The 12 shared pathways with the highest ALS enrichment scores are all translation and ribosome terms. The three highest are L13a-mediated translational silencing (NES 3.15 in ALS, 2.69 in PD dopaminergic neurons), GTP hydrolysis and joining of the 60S ribosomal subunit (3.14 and 2.70), and SRP-dependent cotranslational targeting to membrane (3.12 and 2.52). The Reactome response of *EIF2AK4* (GCN2) to amino acid deficiency [20] ranks eleventh of the twelve on the ALS side (3.01 and 2.67) and is the only member of the block that names a stress kinase (Figure 4b). Two qualifications attach to the PD side of the comparison. The first is compositional, and it is the same one that prevents a verdict on the axonal panel below: across the 17 donors the ribosomal score falls as dopaminergic content rises (ρ = −0.468, *p* = 0.058), and disease donors carry less dopaminergic content systematically, so the block is measured in a compartment whose composition differs between groups. The second is annotational: for the reason given above, translation panel values from the two PD resources are not comparable on an absolute scale, although the 86-pathway comparison itself runs between GSE153960 and GSE178265, where all 14 panel members are present, and is unaffected.

The axonal transport motor panel diverges between the diseases, but composition prevents a verdict in PD. Against median Wald statistics of −5.38 in ALS cervical cord (*p* = 3.6 × 10^−4^) and −5.64 in lumbar cord (*p* = 4.5 × 10^−5^), PD dopaminergic neurons return +1.41 (*p* = 0.082) and PD whole tissue −0.27 (*p* = 0.27) (Figure 4c). The panel is measurable in neuronal nuclei: across the ten panel genes, the median per-gene detection rate in GSE157783 is 85.8% of annotated dopaminergic and 75.2% of excitatory nuclei, against 8.6% of microglia, so the positive PD value is not a dropout artefact (Figure S5d). It is, however, not separable from dopaminergic content. Across the 17 donors, the axonal score falls as the marker-high fraction rises (ρ = −0.525, *p* = 0.031). The estimate keeps its sign and size within the eight control donors alone, where no disease effect can contribute (ρ = −0.476, *p* = 0.233, *n* = 8; Figure 4c). The ribosomal and mitochondrial scores show the same dependence (ρ = −0.468, *p* = 0.058 and ρ = −0.395, *p* = 0.117), so the confound is not specific to the axonal panel. Disease donors carry less dopaminergic content systematically, so any panel intrinsically lower in dopaminergic content than in neighbouring neurons will rise in disease for that reason alone. Restricting to donors matched on content (marker-high fraction ≤ 0.26) leaves a disease difference in place (axonal score +0.350 in PD versus −0.510 in controls) but retains only three controls, too few to decide. The ALS transport deficit is established across two segments, two cohorts, and nine of ten models; the corresponding question in PD dopaminergic neurons is not adjudicable with these data.

The shared stress-response block is likewise not localised to the dopaminergic compartment. Dopaminergic and non-dopaminergic nuclei were compared within the same donors (Section 4.6). The 695 leading-edge genes pooled over all 86 shared gene sets score lower in the dopaminergic compartment (Δ = −0.054, Wilcoxon *p* = 2.6 × 10^−3^, 11 of 15 donors). The 663 leading-edge genes specific to ALS run the other way (Δ = +0.011, *p* = 3.4 × 10^−3^, 14 of 15 donors), which rules out a generic compartment-wide offset (Figure 4d). Across the 12 annotated cell types of GSE157783, the recoverable part of the same leading edge is likewise lowest in the annotated dopaminergic neurons; only relative ordering is interpretable there, since 73 of the 695 genes are absent from that deposit (Figure S5e). A donor-level test agrees. Mean translation scores separate neither group across all selected nuclei (+0.087 in PD versus +0.028 in controls, *p* = 0.28, *n* = 17 donors) nor within the dopaminergic compartment alone (−0.038 versus −0.027, *p* = 0.61), while the same test does separate the groups on dopaminergic content (*p* = 0.046). The shared GCN2 and translation block is thus a gene-set, rank-level property of the two diseases, not a demonstrated per-donor elevation of translational output inside PD dopaminergic neurons.

## 3. Discussion

In these data, the ALS and PD contrasts overlap in two places: a complement and phagocytosis program measured at compartment level in ALS cord and in PD whole midbrain tissue, and a shared block of translation and ribosome gene sets. The microglial attribution of the first comes from a localisation test that carries no diagnosis term, and within PD microglial pseudobulk the same panel is null (median Wald +0.21, *p* = 0.57). The translation block includes the Reactome response of *EIF2AK4* (GCN2) to amino acid deficiency [20], which ranks eleventh of the twelve strongest shared sets on the ALS side (NES 3.01 in ALS, 2.67 in PD) and is the only member that names a stress kinase. No measurement of GCN2 activity, eIF2α phosphorylation, or ATF4 output exists in these resources, so the block is named by annotation rather than by demonstrated kinase dependence. The microtubule transport deficit that dominates the ALS cord is established on the ALS side only; the corresponding measurement in PD dopaminergic nuclei is not separable from dopaminergic content, so these data neither establish nor exclude an ALS-like deficit there (Section 2.5).

The complement axis is a compartment-level property of the tissue: the same five microglial transcripts rise in both ALS cord segments and in PD whole midbrain tissue (Section 2.4; Figure 3f). Cross-disease agreement at gene level is highest in microglia and in whole tissue and weaker in astrocytes, but it separates from chance in neither neuronal compartment nor in oligodendrocytes, so the ordering is not a glia-versus-neuron split (Figure 3e). What places the signal is a localisation rather than a contrast: a 200-gene signature defined in bulk ALS cervical cord scores highest on microglia in every one of the 11 midbrain donors of GSE157783—6 of them controls—at a donor mean of +3.55 (s.d. 0.536) against +0.73 for the next class. This ranking states which cells carry these transcripts, not that a diagnosis raises them. Neither axis is a measurement of transcription per cell.

The obvious objection to that placement is circularity: a signature raised in bulk ALS cord will contain glial activation genes, so microglia are where it must land. The data answer this in two parts. The concentration is real and is not what a comparable gene set gives—111 of the 190 signature genes present in the midbrain deposit peak in microglia, against 10.3% expected from expression-matched random sets and 10.5% of all expressed genes, a 5.65-fold excess that no permutation of 10,000 reached. But the microglia-first ordering does depend on those genes: removing all 111 leaves 79 on which astrocytes rank first and microglia fourth. What survives that removal is narrower than a clean glial-versus-neuronal split: the five highest compartments on the reduced score are all non-neuronal, and no neuronal compartment is positive, but oligodendrocytes and oligodendrocyte precursors are negative too, so the residual signal is astrocytic and vascular rather than glial as a class. The claim the reduced score supports is therefore that the shared axis is not neuronal; which non-neuronal compartment carries it depends on a gene membership that is itself 5.65-fold concentrated in microglia.

The batch structure of the ALS resource is a result of this study, not a footnote to its methods. Contributing project—NYGC ALS Consortium versus Target ALS Post-mortem Core [15,16]—is a second batch variable that tracks diagnosis more strongly than library chemistry does, and adjusting for it removes about a third of the naive differential expression (Section 2.1; Figure 1b,c). In lateral motor cortex, medial motor cortex and occipital cortex, the association is complete separation: every ALS sample comes from one project, so disease and project effects are not jointly identifiable, and 208 ALS and 48 control samples were removed. This exclusion is costly, because motor cortex is what an ALS study most wants. What survives the adjustment is glial activation in the ALS cord, in agreement with the prior report from this consortium resource [15]; the contributing project is therefore a property of GSE153960 that any reuse of it has to model.

The ALS side is dominated by microtubule-based transport. Vesicle transport along microtubules is the most strongly depleted gene set in the cervical cord, and the lumbar cord names retrograde and anterograde axonal transport directly (Section 2.3; Figure 2c). *KIF5A*, an established ALS gene [7], falls by 0.34 ± 0.05 log_2_ units in cervical and 0.39 ± 0.07 in lumbar cord, and the dynactin subunit *DCTN1*, mutated in motor neuron disease [9], by 0.26 ± 0.04 and 0.31 ± 0.04. Both are significant in both segments, but both fall short of the |log_2_ fold change| > 0.5 threshold used to call differential expression here (−0.34 and −0.26 in cervical cord), so they enter as effect sizes rather than as threshold calls (Section 2.2). Transport failure precedes symptoms in ALS models, though it can also dissociate from degeneration [12,14].

Because ALS is a distal axonopathy [5], composition is the competing explanation, and this design does not exclude it. Only the two M3 fits carry a composition term at all; the remaining eight are nested or stratified designs without one, and that term is a proxy derived from the matrix it adjusts (Section 4.3). After adjustment the pan-neuronal panel no longer separates from the protein-coding background (*p* = 0.278), but it still sits at a median Wald of −2.25, larger in magnitude than the −1.68 of the structure-and-transport panel in that same composition-adjusted fit (Figure S3c), which carries the neuronal-content term without the project term and is therefore not the final model, under which the structure-and-transport panel reaches −2.07, so a transport-specific depletion is not cleanly separable from residual neuronal loss in bulk cord. No ALS-side analogue of the dopaminergic-content regression that voids the PD verdict is available in these records. Nor does the regeneration-associated panel decide the question. It reaches significance in none of the ten fits (median Wald +1.66, *p* = 0.108 in cervical cord under the final model); only a nine-gene variant that omits *KLF7* (Wald −2.80) crosses the threshold at all (*p* = 0.044), and dropping any one of *CREB1*, *GADD45A*, *SMAD1*, *KLF6*, or *GAP43* from that variant returns it above threshold. The structure-and-transport depletion itself reproduces in nine of the ten fits, failing only in the NYGC lumbar stratum (−0.34, *p* = 0.134), but replication cannot separate it from neuronal loss: that confound acts in every one of the ten fits alike, and in the composition-adjusted comparison above the pan-neuronal panel is the more negative of the two. The reservation we attach to the PD axonal panel therefore attaches here as well.

The shared complement axis rests on established biology that our data constrain in a specific way. Complement tags synapses for microglial engulfment in development and in disease [23,24], the *TREM2*–*TYROBP* module is the transcriptional core of the disease-associated microglial state [21,22], and reactive microglia are documented in PD nigra and in ALS cord [25,26]. What these data do not show is induction per microglial cell. Inside PD microglial pseudobulk, the complement and phagocytosis panel is null (median Wald +0.21, *p* = 0.57), so a compartment-level rise with a flat within-microglia test fits a larger microglial population as readily as higher expression per cell. The same caution applies to the ALS arm, whose only composition covariate is a pan-neuronal proxy: neuronal loss raises the glial share arithmetically, and bulk cord cannot separate that from induction either. The donor-level projection does not settle it. This test carries no diagnosis term—microglia rank first in the 6 control donors as readily as in the 5 PD donors, and *p* = 9.8 × 10^−4^ is the floor of the test at 11 donors—so it identifies the compartment in which these transcripts are expressed in midbrain tissue and says nothing about disease. Localisation and induction are separate questions, and neither arm of this study answers the second.

The shared translation block includes the GCN2 arm of the integrated stress response, and it carries a compositional confound of the same weight as the one that voids the axonal verdict. GCN2 senses uncharged tRNA and stalled ribosomes and converges with the other eIF2α kinases on one translational brake [20,27], and pharmacological modulation of that brake alters translational output and rescues TDP-43 toxicity in ALS models [28,29]. This study measured none of that: no assay of GCN2 activity, eIF2α phosphorylation or ATF4 output exists in these resources, so the literature is not a follow-on from the present result. Our evidence is enrichment in ranked lists, measured on the PD side in a compartment whose composition differs between groups. Across the 17 donors of the enriched selection, the ribosomal score falls as dopaminergic content rises (ρ = −0.468, *p* = 0.058)—the same variable, at nearly the same strength, as the dependence for which we withhold a verdict on the axonal panel (ρ = −0.525, *p* = 0.031)—so the same discount applies.

Two results keep the claim narrow. Donor mean translation scores separate the groups neither across the selection nor inside the dopaminergic compartment. The leading-edge genes of the shared block score lower inside that compartment than in neighbouring nuclei of the same donors, while the leading edge specific to ALS runs the other way, which rules out a generic compartment-wide offset (Section 2.5; Figure 4d). The ten sets that outrank the GCN2 term on the ALS side are undifferentiated ribosome and cotranslational-targeting terms, so what this comparison establishes is a shared rank-level enrichment of translation annotation in two tissues, not a rise in translational output inside PD dopaminergic neurons.

Whether an ALS-like transport deficit exists in PD dopaminergic neurons is not adjudicable with these data. The transport motor panel is strongly negative only in the ALS cord, and in the PD dopaminergic compartment it is positive rather than absent (median Wald +1.41, *p* = 0.082). The panel is measurable in neuronal nuclei of the second midbrain dataset—median per-gene detection 85.8% of annotated dopaminergic and 75.2% of excitatory nuclei against 8.6% of microglia in GSE157783, a descriptive comparison made in a different resource from the one in which +1.41 was measured, and one resting on the 74 annotated dopaminergic nuclei that deposit holds (Section 2.5; Figure S5d). It does not establish that the GSE178265 estimate is free of dropout. The estimate is in any case inseparable from composition. The donor-level axonal score falls as the marker-high fraction rises (ρ = −0.525, *p* = 0.031) and keeps its sign within the eight control donors alone (ρ = −0.476, *p* = 0.233, *n* = 8), where no disease effect can contribute, though at that number of donors the estimate is not significant. Disease donors carry less dopaminergic content, and restricting to content-matched donors leaves the difference in place (axonal score +0.350 in PD versus −0.510 in controls) on three control donors, too few to decide. Any panel intrinsically lower in dopaminergic than in neighbouring neurons rises in disease for that reason alone. Axonal arbour size is central to arguments about dopaminergic vulnerability [4], so the question deserves a design built to answer it; ours cannot, and we claim neither an ALS-like deficit in PD nor its absence.

Neither axis is nominated as a therapeutic target by these data. *TREM2* has an active target-development literature in Alzheimer’s disease [30,31,32]. None of it bears on what this study measured: *TREM2* enters here only as one of five transcripts in bulk contrasts that carry no glial-content covariate, and in a localisation test that carries no diagnosis term. What this study contributes is placement: which compartment carries these transcripts in PD midbrain. It establishes neither per-cell induction, nor specificity against tissue degenerating for other reasons, nor a direction of intervention: what these data place is a compartment, and microglial mutant *SOD1* expression sets the rate of disease progression in mouse ALS without telling us the sign of an intervention in human tissue [33].

Three additions would decide what this design leaves open. The first is anatomical control: a third neurodegenerative disease in a matched region, and a non-degenerative injury control, are needed before a shared axis can be called disease-specific rather than a general property of degenerating tissue. The second is resolution on the ALS side: separating microglial expansion from per-cell induction requires a glial-content covariate these records lack, or single-nucleus data from ALS cord at donor scale. The third is composition-matched sampling of dopaminergic neurons, with enough donors to survive stratification by content. The defects each of these would remove are set out below.

### Limitations

Anatomical region and diagnosis are completely confounded in the cross-disease comparison. Our ALS contrast is spinal cord, and our PD contrast is midbrain, so every statement about what the two diseases share is also a statement about what these two tissues share, and this design cannot separate the two readings. The three cortical regions in which the contributing project was completely separated from diagnosis were removed from differential expression (Section 4.3). Thoracic cord draws both arms almost entirely from Target ALS (ALS 42/42, controls 9/10; χ^2^ *p* = 0.43), so the contributing project carries no contrast there; it enters no analysis here, and the ALS arm therefore speaks for cervical and lumbar cord alone. Of the 1242 samples in the primary analysis set, the 379 of cervical and lumbar cord (295 ALS, 84 control) are the ones that enter a disease contrast. Cerebellum and frontal cortex contribute more ALS samples than either cord segment but were set aside on anatomical grounds rather than on any property of their results, and the ALS arm is correspondingly a spinal cord result and not a survey of the central nervous system. Nor is there a control for the class of the insult. GSE153960 contributes no other neurological-disease sample to cervical or lumbar cord (Table S1). Such samples exist in three regions: frontal cortex (*n* = 45), cerebellum (*n* = 49), and temporal cortex (*n* = 35). For the two whose contributing-project composition is recorded here, those samples come entirely from one project—frontal cortex 45/45 and cerebellum 49/49 from the NYGC ALS Consortium—so neither region can serve as a disease control against groups drawn from both projects. The contributing-project composition of the 35 temporal-cortex samples was not assembled here, so whether they could serve that purpose is undetermined. No non-degenerative injury and no third neurodegenerative disease therefore enter as a comparison arm. A microglial complement and phagocytosis response is reported across degenerating central nervous system tissue [21,22,23], and no analysis here asks whether that axis is absent from tissue degenerating for another reason, so its specificity to these two diseases is untested.

The ALS arm carries no covariate for glial content. The pan-neuronal proxy of Section 4.3 is the only composition term these records support, and it is derived from the matrix it adjusts. Loss of neurons lowers the neuronal share of the cord and raises the glial share, so *TREM2*, *TYROBP*, *C1QB*, *C3*, and *CD68* can rise in bulk cord with no change in expression per glial cell. We apply the reservation equally to both arms: in PD, the within-microglia test is null (median Wald +0.21, *p* = 0.57, 3903 nuclei from five PD and six control donors), and on the ALS side, no within-microglia measurement exists at all. Neither arm establishes induction of complement per glial cell. Nor is the compartment attribution independent of which genes define the signature: the microglia-first ranking is carried by the 111 signature genes that peak in microglia, and the 79 remaining genes rank astrocytes first, so only the glial-versus-neuronal contrast is robust to that membership.

The dopaminergic compartment is enriched rather than pure, and its composition differs between the groups compared in it. Of the 55,103 selected nuclei, 13,703 reach the dopaminergic marker score of five, and the 12,973 nuclei (23.5% of the selection) that form the dopaminergic compartment are those falling in the 11 Leiden clusters in which at least half the nuclei do so; that compartment captures 92.8% of the marker-high nuclei. Disease donors carry less dopaminergic content than controls (marker-high fraction 0.124 versus 0.340, Mann–Whitney *p* = 0.027, *n* = 17 donors). Donor-level panel scores track that fraction: axonal ρ = −0.525 (*p* = 0.031), ribosomal ρ = −0.468 (*p* = 0.058), and mitochondrial ρ = −0.395 (*p* = 0.117). This is why we offer no verdict on axonal transport in PD, and the same reservation attaches at nearly the same strength to the shared translation and GCN2 block, which is measured in the same compartment and moves with the same variable. The composition-matched subset (marker-high fraction ≤ 0.26) leaves a disease difference in place (axonal score +0.350 in PD versus −0.510 in controls) but retains three control donors, too few to decide either way. The deposited diagnosis field of GSE178265 records only Ctrl and Disease [18], so its disease group is a nigral degeneration group in which Parkinson’s disease is not separated from dementia with Lewy bodies.

Power on the PD side is small. GSE157783 supplies five PD and six control donors [17], and the donor rather than the nucleus is the unit of replication [19], so its cell-type contrasts carry no capacity for covariates, and a null within them is not evidence of absence. The donor-level signed-rank test reaches *p* = 9.8 × 10^−4^ because that is the smallest two-sided value available at 11 donors, not because the effect is bounded more tightly. The curated panel statistics are uncorrected, are computed on panels that are subsets of their own backgrounds, and can turn on membership, as the regeneration-associated panel does. This leave-one-out test was run on the regeneration-associated panel alone; the transport, mitochondrial and neuroinflammation panels were not subjected to it, so their own dependence on membership is unmeasured here.

Post-mortem records limit the ALS arm further. RNA integrity, donor age, sex, and post-mortem interval are absent from the GSE153960 metadata, so no ALS contrast is adjusted for them and no sensitivity analysis for transcript-length bias is possible. Degradation is not uniform across transcripts: degradation rate rises with coding-sequence length, 3′ UTR length, and GC content, although total transcript length is not itself a predictor [34]. Neither RNA integrity nor a transcript-length distribution is recoverable from these records, so a degradation-related contribution to the transport and neurofilament result can be neither excluded nor quantified here. The age, sex, and post-mortem-interval balance of the 11 GSE157783 donors is likewise not established here.

The ALS cohort is genetically heterogeneous, and this study does not stratify on it. GSE153960 records no genotype, so sporadic and genetic cases enter the same arm. De-identified clinical metadata released for the same NYGC spinal cord samples record a reported mutation in 65 of 261 ALS samples (24.9%), with *C9orf72* in 49, *SOD1* in 8, *FUS* in 4, *OPTN* in 2, and *ANG* in 2; limb onset in 166 and bulbar onset in 70; and a median disease duration of 3 years [15]. Those identifiers cannot be joined to individual samples, so these figures describe the resource rather than this analysis set, but they set the scale of the heterogeneity that the ALS effect estimates reported here average over. Disease stage, cause of death, and diagnostic criteria are likewise unrecorded in these files and differ between the three studies. The same released metadata put RNA integrity higher in ALS than in control samples of this resource, an imbalance whose direction would inflate rather than deplete long-transcript signal in the disease group and therefore cannot generate the axonal transport depletion reported here.

Platform varies with disease and with dataset and cannot be separated from either. The library preparation method is associated with diagnosis within GSE153960 and is modelled in every fit, but sequencing platform is not among the recorded fields and is not modelled. Across the three resources, the platforms differ entirely, so the cross-disease comparison confounds platform with diagnosis as completely as it confounds anatomical region with it. The two arms also measure different things: bulk RNA-seq of whole cord against single-nucleus RNA-seq of dissociated midbrain. A change in cellular composition and a change in expression per cell produce the same bulk signal, which is why every complement and phagocytosis statement in the ALS arm is compartment-level; conversely, single-nucleus data under-represent cytoplasmic transcripts, so the two arms do not sample the same molecular compartment even where they name the same gene set. Cell-type annotation is a third axis of partial comparability: GSE157783 carries the depositors’ twelve-class annotation, GSE178265 is used through the marker- and sorting-based selection defined in Section 4.6, and GSE153960 carries no cell-level annotation at all, so panels are realised on different memberships and are read within dataset only.

One panel reported here does not survive covariate adjustment. The mitochondrial panel reaches a median Wald statistic of +3.52 in cervical cord and +2.14 in lumbar cord under the primary model, but returns +0.83 and +0.84 when the same panel is computed on an independently published analysis of these spinal cord samples that carries donor age, sex, RNA integrity, and genotype principal components [15]. The axonal transport, complement and phagocytosis, neuroinflammation, and translation panels reproduce on that analysis, and the transport, complement, and translation results are also not explained by transcript length under length-matched permutation (Table S8), but the mitochondrial reading is not carried forward.

All three resources are cross-sectional end-stage tissue. No ordering in time is recoverable, so whether the transport deficit precedes or follows degeneration, and whether the microglial program drives neuronal loss or records it, lies outside what this design can address.

## 4. Materials and Methods

### 4.1. Datasets

Three public human post-mortem datasets were analysed, all retrieved from the Gene Expression Omnibus (GEO) [35,36]. No new human material was collected, and no controlled-access data were used.

GSE153960 supplies the ALS arm. It is post-mortem bulk RNA-seq contributed jointly by the New York Genome Center (NYGC) ALS Consortium and the Target ALS Human Post-mortem Tissue Core [15,16]. The distributed count matrix holds RSEM expected counts for 58,929 genes across 1659 sample columns; the series itself registers 1838 GSM records. Quantification was performed by the data generators: reads were trimmed with Trimmomatic v0.36 [37], aligned to hg38 with STAR v2.7.2a [38], quantified with RSEM v1.3.1 against GENCODE v30 [39,40] and aggregated with tximport v1.12.0 [41]. This pipeline was not repeated here. Its versions are reported because the low-expression filter and the Wald-statistic ranking used below inherit its properties.

GSE157783 supplies midbrain single-nucleus RNA-seq [17]. The deposited series comprises 41,435 nuclei annotated by the original authors into twelve cell classes, with 26,737 genes in the deposited matrix; its sample records assign five idiopathic PD donors (IPD1–IPD5) and six controls (C1–C6), whereas the source publication describes six patients and five controls. The donor assignment used throughout this study is the one recorded in the GEO deposit. Median sequencing depth is 5293 unique molecular identifiers and 2438 detected genes per nucleus; 22,433 nuclei come from controls and 19,002 from PD donors. Class sizes are strongly unequal, from 21,268 oligodendrocytes down to 120 *CADPS2*-positive neurons and 74 annotated dopaminergic neurons, and this inequality determines which classes are analysable (Section 4.5).

GSE178265 supplies single-nucleus RNA-seq of substantia nigra pars compacta [18]. Raw human sequence is restricted through dbGaP, but the processed matrix is public and is the version used here: 434,340 nuclei × 41,625 genes, 1654,688,953 non-zero entries, aligned to hg19. The human portion comprises 97 libraries from 22 donors—90 from substantia nigra and 7 from caudate nucleus, the latter all controls—and the fluorescence-activated cell sorting (FACS) classification field records 51 positive, 45 negative, and 1 NeuN library. The GSM characteristics supply tissue, sex, age, post-mortem interval, disease, status, cause of death, FACS classification, 10x chemistry version, and genome build. The disease field takes only two values, Ctrl and Disease. Parkinson’s disease therefore cannot be separated from dementia with Lewy bodies within this dataset. This is a property of the deposited annotation; it is not resolvable from the public files, and it is carried through to the interpretation rather than treated as resolved.

Genome builds differ across resources: hg38 with GENCODE v30 for GSE153960, hg19 for GSE178265. Every cross-dataset comparison in this study is therefore made on gene symbols and never on coordinates or transcript models.

### 4.2. Metadata Join and Sample Selection in GSE153960

Sample metadata were joined to matrix columns on the GSM characteristics field sample id alt, whose values carry the matrix column identifiers (CGND-HRA-XXXXX, with a -2 suffix on re-sequenced libraries). Matching used exact strings, since the -2 suffix forms part of the metadata value and stripping it misassigns re-sequenced libraries. Exact matching resolved 1639 of the 1659 matrix columns. The 20 unresolved columns have no corresponding GEO record and were discarded. One value, CGND-HRA-01288-2, is carried by two GSM records, the same specimen sequenced under both the Manual and the Automated library protocol, so the single matrix column cannot be attributed to one library and was excluded rather than assigned arbitrarily.

The per-sample fields retained from the join are anatomical region, diagnostic group, subject identifier, library preparation method, and contributing project (Table S1). Donor age, sex, post-mortem interval, and RNA integrity are absent from these records and could not be modelled. The public record was searched exhaustively for them. The 1838 GSM entries of GSE153960 carry six sample characteristics—contributing project, tissue, subject identifier, diagnostic group, matrix column identifier, and library preparation method—and the series description directs further metadata requests to the contributing consortium. Extending the search to the parent SuperSeries GSE137810, whose other constituent series do record donor sex and age, recovers sex for 17 and age for 7 of the 215 donors of the spinal cord analysis and RNA integrity for none, which is too sparse to support a covariate. The primary analysis set is the 1242 samples belonging to the two comparison groups: 962 samples from 217 donors with ALS-spectrum motor neuron disease, and 280 samples from 102 donors with no neurological disease. Samples carrying an additional or alternative neurological diagnosis were held out of the primary comparison. The two spinal regions analysed contribute 155 ALS and 41 control samples (cervical cord) and 140 ALS and 43 control samples (lumbar cord). Per-sample use and exclusion reasons for all 1639 joined samples are given in Table S1.

Every ALS comparison in this study is therefore unadjusted for age, sex, post-mortem interval, and RNA integrity, while the PD dopaminergic comparison of Section 4.6 carries sex, age, and post-mortem interval in its third model. RNA integrity is the consequential omission, because RNA degradation rates covary with coding-sequence length, 3′ UTR length, and GC content [34], so a contribution from differential RNA quality to the axonal-transport result cannot be excluded from the ALS arm by covariate adjustment. No proxy for it is recoverable from these records. Cohort-stratified and cross-segment refits are reported in its place (Section 4.4), as reproducibility of the estimate rather than as a bound on RNA quality.

### 4.3. Differential Expression

RSEM expected counts were rounded to the nearest integer and analysed with PyDESeq2 v0.5.4 [42]. A gene was retained for testing if its rounded count reached 10 in at least as many samples as half the size of the smaller group of that comparison—20 samples in cervical cord and 21 in lumbar cord, counted across all samples of the comparison—which left 21,506 genes testable in cervical cord and 21,540 in lumbar cord. Differential expression was declared at Benjamini–Hochberg adjusted *p* < 0.05 [43] together with |log_2_ fold change| > 0.5. Both criteria were applied uniformly to every comparison in the study, including the PD comparisons, so that all gene counts are produced by one rule.

Donor independence was enforced by construction rather than by a random-effect term. Each anatomical region was modelled separately, and within a region, a donor contributing more than one sample was reduced to a single sample, so no donor appears twice in any fitted model. The donor structure of GSE153960 permits this: the median donor contributes 4 samples and the maximum 9, distributed across regions, and 77 donors contribute a single sample in total. A donor random intercept is therefore not identifiable in any model fitted here, since each of the 196 cervical and 183 lumbar donors contributes exactly one sample to its region. Fitted instead on the joint two-segment design, in which 164 donors contribute to both, the random intercept absorbs a substantial share of the residual variance (median intraclass correlation 0.706) and leaves the disease effect unchanged: Pearson r = 0.998 against the fixed-effect estimate, with no sign reversal among genes significant under either model (Table S8).

Two technical variables are associated with diagnosis in this resource, and they define the model specification. Library preparation method is associated with diagnosis across the primary analysis set (χ^2^ *p* = 6.1 × 10^−5^). The contributing project is associated with it far more strongly (χ^2^ *p* = 1.12 × 10^−22^), and that association persists within regions (*p* = 2.88 × 10^−3^ in cervical cord, *p* = 7.67 × 10^−4^ in lumbar cord, and *p* = 6.24 × 10^−5^ in cerebellum). Project and library preparation are themselves associated (χ^2^ *p* = 3.87 × 10^−69^) but are not the same axis, so adjusting for chemistry alone leaves project uncontrolled (Figure S1a,b).

The primary ALS contrast was restricted to the cervical and lumbar spinal cord before any model was fitted. The question this study asks is about the compartment that degenerates first in ALS, and the spinal cord is where the motor neurons and their proximal axons are; the remaining regions of GSE153960 are either not primary targets of the disease, as with the cerebellum, or are cortical regions whose provenance structure is described next. Region-wise fits for six regions are reported in Figure S2a as a description of detection power and are not interpreted as disease effects. Three regions were excluded from differential expression a priori: lateral motor cortex, medial motor cortex, and occipital cortex. In each of them, 100% of ALS samples derive from a single project (82/82, 81/81, and 45/45; χ^2^ *p* = 1.27 × 10^−6^, 3.30 × 10^−5^, and 3.33 × 10^−5^), so project is completely separated from diagnosis. Disease and project effects are not jointly identifiable there under any model, so no effect estimate from those regions is interpreted or carried into any cross-disease comparison; the counts plotted in Figure S2a are shown only to document how detection tracks control-group size. For the same reason, the other neurological-disease samples were not used as a disease control group: in the two regions we examined, they come entirely from one project, and in the frontal cortex entirely from one library preparation as well, with 91.8% of the cerebellar samples drawn from that same preparation (Figure S2c,d).

Three nested designs were fitted to each analysed spinal region: M1, ~libprep + condition; M2, ~project + libprep + condition; and M3, ~project + libprep + neuro_score + condition. M3 is the primary model for all ALS results. All three are reported side by side throughout, because the difference between them is itself informative about how much of the ALS signal is carried by technical structure and by cellular composition.

The neuronal score is a within-dataset proxy for neuronal content. It is the sample-wise mean of log1p-transformed counts per million across a pan-neuronal marker panel—*SNAP25*, *SYT1*, *SYP*, *RBFOX3*, *ENO2*, *UCHL1*, *SYN1*, *NRGN*, *CAMK2A*, and *GRIN1*—standardised to zero mean and unit variance within the samples of that region. A twelve-gene variant of the same panel, which additionally contains *NEFL* and *STMN2* (pan_neuronal_composition_proxy_12 in Table S2), is used for the descriptive between-group contrast and is the panel plotted before and after adjustment in Figure S3b,c; the ten-gene covariate panel is listed there as pan_neuronal_composition_covariate_10. The covariate is included because ALS degenerates motor axons and then motor neuron cell bodies in a dying-back sequence [5], which shifts the cellular composition of the cord, and that shift is directly measurable in the motor-neuron-restricted markers reported in the Results (Figure S3a). The twelve-gene variant was tested against the protein-coding background of Section 4.8 before and after adjustment, and both values are reported in the Results (Figure S3c). The proxy is derived from the same matrix it adjusts, so it necessarily removes some neuron-intrinsic disease signal along with composition. M3 is therefore not treated as a composition-free estimate, and every panel result is reported across M1, M2, and M3 so that the size of that removal is visible. Because the genes that build a covariate cannot serve as an independent control for the model that covariate adjusts, the primary model was refitted with the composition term rebuilt from ten pan-neuronal genes sharing no member with any panel tested here (*MAP2*, *SYN2*, *SYT4*, *NSF*, *ATP1A3*, *CPLX1*, *CPLX2*, *VAMP2*, *STX1A*, and *MYT1L*), and with that term entered as a quadratic and as an orthonormal cubic basis. The disjoint refit agrees with the primary model at Spearman ρ = 0.998 in cervical and 0.9997 in lumbar cord over the Wald statistics of all tested genes, the two non-linear refits at ρ ≥ 0.997, and every panel statistic is reproduced (Table S8).

The pan-neuronal proxy is the only composition covariate this resource supports. No covariate for glial content was fitted, and none is recoverable from these records. Loss of neurons lowers the neuronal share of the tissue and raises the glial share, so a rise in microglial transcripts in bulk cord can follow from a change in composition alone, with no change in expression per glial cell. The complement and phagocytosis statistics of Section 4.8 are accordingly compartment-level properties of the tissue in both diseases, and this design does not resolve them into expression per glial cell in either arm.

### 4.4. Cohort-Stratified Analysis

Each ALS result was additionally refitted within each contributing cohort separately, and the number of fits in which it holds is reported with it rather than used as a filter. The design ~libprep + condition was refitted separately within each stratum, the project term being constant within a stratum and therefore dropped. The NYGC stratum holds 63 ALS versus 28 controls in cervical cord and 61 versus 32 in lumbar cord; the Target ALS stratum holds 92 versus 13 and 79 versus 11. The low-expression filter, the significance rule and the donor-independence constraint of Section 4.3 were applied unchanged within each stratum.

Agreement between strata was quantified in three ways. The first is Spearman rank correlation of log_2_ fold changes over genes tested in both strata. The second is directional agreement, computed only among genes reaching adjusted *p* < 0.05 in both. The third is the number of gene sets reaching FDR < 0.05 in both after rerunning the enrichment analysis of Section 4.7 independently within each stratum. The same three comparisons were applied between cervical and lumbar cord under M3 to test anatomical reproducibility.

Numbers of differentially expressed genes are not compared between strata or between regions anywhere in this study. The strata differ substantially in size, and detection is power-limited: across regions, the number of differentially expressed genes tracks the size of the control group (Spearman ρ = 0.943; Figure S2a). Occipital cortex, which holds the smallest control group, returns no differentially expressed genes at all, at a minimum adjusted *p* of 0.135 over the 20,704 of its 20,732 tested genes that survive independent filtering and carry a *p* value (Figure S2b). Only rank concordance, direction of effect, and panel-level and pathway-level statistics are compared across strata and regions.

### 4.5. Pseudobulk Aggregation for Single-Nucleus Data

The donor, not the nucleus, is the unit of replication in every single-nucleus analysis reported here. Nuclei from one donor are not independent observations of a disease state, and treating them as such inflates the false discovery rate [19]. Unique molecular identifier counts were therefore summed within each donor × cell-type combination before any statistical test, and all differential testing was performed on these donor-level pseudobulk profiles with PyDESeq2 under the filter and significance rule of Section 4.3.

For GSE157783, a cell type was analysed only if it contributed at least 10 nuclei in a donor and reached that threshold in at least three donors per group. Ten of the twelve annotated classes qualified, with between 8583 and 17,313 genes testable depending on cell type; for GABA and ependymal cells, five rather than six control donors qualify, because control donor C3 contributes 4 and 6 nuclei to those classes (Figure S4b). The annotated dopaminergic neurons (74 nuclei, median 5 per donor) and *CADPS2*-positive neurons (120 nuclei) fail these thresholds and were excluded as unanalysable rather than analysed with a caveat. This exclusion is the reason a second, dopaminergic-enriched dataset was required (Section 4.6). Each admitted cell type was modelled as ~condition. Donor age, sex, and post-mortem interval were not modelled in this dataset, and their balance across the 5 disease and 6 control donors is not established here, so these contrasts are unadjusted for donor characteristics, unlike the GSE178265 contrast of Section 4.6. With 5 donors against 6, the contrast has no capacity to carry additional covariates, and it is used for compartment placement and for direction of effect rather than for effect size. A whole-tissue profile was additionally constructed by summing across all cell types within each donor, giving an axis that can be set against the bulk ALS data without a resolution mismatch.

Both single-nucleus datasets were re-embedded from the deposited matrices without donor, batch or covariate correction, so that residual structure would remain visible rather than concealed. GSE157783 and the dopaminergic-enriched selection of GSE178265 were embedded with Scanpy [44,45]. Counts were normalised to 10,000 per nucleus, log-transformed, restricted to the 2000 most variable genes, scaled to unit variance with values clipped at 10, and reduced to 50 principal components. The embedding is the Scanpy implementation of uniform manifold approximation and projection [46] over a 15-nearest-neighbour graph, and the donor structure it retains is quantified in Figure S5a. Clusters were called on the same 15-nearest-neighbour graph with the Leiden algorithm [47] as implemented in Scanpy; they describe the connectivity and marker composition of the dopaminergic-enriched selection and define the dopaminergic compartment used in Section 4.6 (Figure S5b,c). No disease-group comparison is made on an embedding.

Per-nucleus scores were computed with the Scanpy score_genes function, and two kinds are used. The first is the curated panels of Section 4.8. The second is a cross-disease signature taken from the ALS bulk data: the 200 protein-coding genes with the largest positive log_2_ fold change in ALS cervical cord under model M3, among the genes meeting the significance rule of Section 4.3. This signature is defined entirely outside the PD data, and it is the colouring variable of Figure 3a. Signature scores were standardised against control nuclei alone, so that zero is the control mean and one unit is one control standard deviation, and were then averaged within each donor × cell-type combination holding at least ten nuclei. Each cell type was compared against the mean of the same donor across all testable cell types by two-sided Wilcoxon signed-rank test [48] over the 11 donors, with Benjamini–Hochberg adjustment across cell types; cell types represented in fewer than five donors were not tested. The *p* value printed in Figure 3c is the unadjusted value from that test, and 9.8 × 10^−4^ is the smallest value it can return at 11 donors. Dot plots of single genes across cell types report the fraction of nuclei of that cell type carrying at least one count and its mean log-normalised expression, with PD and control nuclei pooled (Figure S6b).

Signature genes absent from a target deposit are dropped before scoring: 190 of the 200 up-signature genes are present in GSE157783, the remaining 10 being absent from the depositor’s matrix. To test whether the compartment on which a signature lands is more concentrated than a comparable gene set would give, each gene was assigned the cell type of its highest mean log-normalised expression across the 12 annotated types, and the signature’s share of genes peaking in that cell type was compared against 10,000 random gene sets drawn from all expressed genes and matched to the signature on decile of mean expression (seed 0). The reported *p* is the empirical share of permutations reaching the observed count. A leave-out variant rescored the signature after removing every gene peaking in the winning cell type.

### 4.6. Dopaminergic Neuron Selection in GSE178265

The selection rule uses no dopaminergic and no axonal genes, since both are measured downstream. Marker scores were computed for each nucleus as summed counts of a marker set normalised per 10,000 unique molecular identifiers; marker set membership is given in Table S2. A nucleus was retained if four conditions held. Its library originated from substantia nigra. This library had been experimentally sorted as *NR4A2*-positive by FACS, which is a recorded property of the library preparation and not a measurement made on the nucleus’s own transcriptome. Its pan-neuronal marker score was at least 30 per 10,000 unique molecular identifiers. Its oligodendrocyte, astrocyte, and microglial marker scores were each at most 2 per 10,000 unique molecular identifiers.

This rule retained 55,103 nuclei from 18 donors. Donors contributing at least 100 nuclei were kept, leaving 17 donors, 9 disease and 8 control, and 27,415 of 41,625 genes passed the low-expression filter after donor-level aggregation. Three models were fitted, labelled P1–P3 to keep them distinct from the ALS cord models M1–M3 of Section 4.3: P1, ~condition; P2, ~log(nuclei) + condition, which is the model used for gene-level reporting; and P3, ~sex + age + post-mortem interval + log(nuclei) + condition. The additional covariates in P3 are balanced between groups in this donor set: Mann–Whitney *p* = 0.596 for age, 0.630 for post-mortem interval, and 0.236 for nuclei per donor, with 6 female and 2 male controls against 4 female and 5 male disease donors. P3 therefore functions as a robustness check rather than as a correction for a known imbalance.

Three checks characterise the selected population, none of which contributed to the selection rule, and their values are reported in the Results (Figure S7a–c). The first is an anatomical contrast between substantia nigra libraries and caudate nucleus libraries, taken from a region that receives dopaminergic projections but contains no dopaminergic somata. The second is a provenance check: the share of marker-high nuclei arising from FACS-positive libraries against the background rate across all nigral libraries. The third is a marker profile: the median dopaminergic, pan-neuronal, oligodendrocyte, astrocyte, and microglial scores of the selected population set against those of the unselected nigral remainder.

One composition diagnostic is defined here because it constrains what the PD panel results can mean. For each donor, frac_DAhigh was computed as the fraction of that donor’s selected nuclei whose dopaminergic marker score exceeds 5 per 10,000 unique molecular identifiers (Table S3) and was compared between groups by two-sided Mann–Whitney test (Figure S7d). Donor-level panel z-scores were then correlated with frac_DAhigh by Spearman rank correlation, computed both across all 17 donors and within the 8 control donors alone (Figure 4c). The within-control correlation asks whether an association exists in the absence of disease. The dopaminergic identity panel is correlated with frac_DAhigh in the same test (ρ = +0.961 across all donors; ρ = +0.833, *p* = 0.010 within controls alone), which sets the scale on which the remaining panel correlations are read. A subset analysis restricted to donors with frac_DAhigh ≤ 0.26 retains only three control donors and is reported without inference.

One further comparison places the shared stress-response block within this selection. The leading edge of the 86 shared gene sets was pooled across sets on both the ALS and the PD side, giving 695 unique genes; the same construction applied to the gene sets significant in ALS alone gave a specificity-control set of 663 genes measurable here. Both sets were scored per nucleus with the Scanpy score_genes function over the 55,103 selected nuclei. Each nucleus was then assigned either to the dopaminergic compartment or to the remainder, that compartment being the 11 Leiden clusters of Section 4.5 in which at least half of the nuclei are marker-high, 12,973 nuclei or 23.5% of the selection (Figure S5b). Donor means were formed separately in the two compartments for the 15 donors contributing at least 30 nuclei to both, and the paired difference was tested by a two-sided Wilcoxon signed-rank test (Figure 4d). The same 695 genes were scored a second time across the twelve annotated cell types of GSE157783 (Figure S5e). Only 622 of them are measurable in that deposit, and 61 of the 73 that are not are cytoplasmic ribosomal proteins (Section 4.8), so the values there are read as a ranking and not as magnitudes.

### 4.7. Gene Set Enrichment Analysis

Gene set enrichment analysis was performed with GSEApy v1.3.1 in prerank mode [49], implementing preranked GSEA [50]. The ranking statistic is the PyDESeq2 Wald statistic rather than the log_2_ fold change, because the Wald statistic incorporates the estimated precision of each effect and therefore does not place low-count genes with large but poorly estimated fold changes at the extremes of the ranking. Every gene surviving the low-expression filter for a comparison entered its ranked list; no significance threshold was applied before ranking. Each run used 1000 permutations, random seed 42, and a gene set size range of 15 to 500 members. Four libraries were queried, all as distributed through Enrichr [51,52]: GO Biological Process 2023 [53,54], KEGG 2021 Human [55,56], Reactome 2022 [57], and MSigDB Hallmark 2020 [58]. Significance is reported at permutation-based FDR < 0.05.

The libraries are keyed on gene symbols while the ALS matrix is keyed on versioned Ensembl identifiers, so identifiers were stripped of their version suffix and mapped through the same GENCODE v30 annotation used for quantification. Where a symbol received more than one Ensembl identifier, the entry with the largest absolute Wald statistic was retained, so that each symbol enters a ranked list exactly once and no gene set is inflated by duplicate members.

Where a term appears in more than one library, the instance with the largest |NES| is retained, so a duplicated term is reported at its most extreme score, and its enrichment score is displaced away from zero relative to a randomly chosen instance. Every gene-set count reported in this study is on a duplicate-collapsed basis and counts unique term names. One term, Apoptosis, is annotated in both KEGG and Hallmark and is significant in both cohort strata under both annotations, so the stratified intersection contains 326 unique terms and 327 term–library pairs. Permutation FDR is controlled within each library and each comparison separately. Counts pooled across the four libraries, and the shared-set intersections of Section 2.2 and Section 2.5, therefore carry no joint error rate; they are reported as descriptive counts of concordance, not as a discovery set with a controlled false-discovery proportion.

Two term-name hazards were resolved by inspecting set contents rather than names. Two terms concern the axoneme: Axonemal Dynein Complex Assembly and Axoneme Assembly. Both describe the ciliary axoneme and not the neuronal axon, and both are excluded from every axonal statement in this work despite reaching strong significance. Regulation of Expression of SLITs and ROBOs is populated largely by ribosomal protein genes in the Reactome 2022 release and is therefore read as part of the translational block rather than as axon guidance. Few axonal terms clear FDR correction in either direction. All gene sets whose names denote axonal processes and survive the axonemal exclusion—28 in cervical cord and 27 in lumbar cord—were therefore extracted regardless of significance. They are summarised as a distribution, by median NES and by the fraction with NES < 0, in the primary model and in both cohort strata.

### 4.8. Curated Pathway Panels and Statistical Comparison

Seven curated panels were assembled from the literature: axonal transport motors (10 genes), axonal structure (10), regeneration-associated genes (10), mitochondrial (17), translation and ribosome (14), neuroinflammation (16), and complement and phagocytosis (10). A combined ten-gene axonal structure-and-transport panel (*NEFL*, *NEFM*, *NEFH*, *KIF5A*, *DCTN1*, *MAPT*, *TUBB3*, *MAP1B*, *TUBA1A*, and *STMN2*) is used for the model-robustness and adjustment comparisons. Three control panels—pan-neuronal, motor-neuron, and dopaminergic identity—are defined alongside them. Full membership and per-dataset availability of every panel are given in Table S2.

For each panel in each comparison, the test statistic is the median PyDESeq2 Wald statistic across the panel members surviving that comparison’s low-expression filter. It was tested by a two-sided Mann–Whitney U test [59] against the Wald statistics of all tested protein-coding genes in the same comparison. The background is restricted to protein-coding genes throughout—16,248 genes in the cervical cord comparison—because every panel member is protein-coding, and because the full tested set is displaced towards negative statistics by its non-coding component, which biases any positively directed panel. The realised size of every panel in every comparison is reported with the statistic, since the tested background differs between comparisons.

No dataset realises every panel in full, and values realised on different memberships are not numerically comparable across datasets or across compartments. Beyond the translation and mitochondrial panels detailed below, the complement and phagocytosis panel is realised on 9 of its 10 members in both PD datasets, and the axon structure panel likewise on 9 of 10. The neuroinflammation panel is realised in 13 of its 16 members in GSE157783 and 12 of 16 in GSE178265. Sparser compartments realise less still, down to 5 of 10 for complement and phagocytosis in PD astrocytes and 4 of 10 in PD excitatory neurons. GSE157783 contains none of the cytoplasmic ribosomal protein genes (*RPL*, *RPS*, *RPLP*, and *RPSA*) and none of the mitochondrially encoded genes, although 76 mitochondrial ribosomal genes are retained. The translation and ribosome panel is therefore realised on 6 of its 14 members there (*EEF1A1*, *EEF2*, *EIF4A1*, *EIF4E*, *EIF2S1*, and *EIF2AK4*), against 14 of 14 in GSE153960 and GSE178265. GSE178265 carries the two ATP synthase subunits under their legacy hg19 aliases *ATP5A1* and *ATP5B* rather than the current symbols *ATP5F1A* and *ATP5F1B*, so symbol-based matching realises the mitochondrial panel at 15 of its 17 members there, against 17 in the other two datasets. Panel values from GSE157783 and GSE178265 are read within the dataset and are not set numerically against the corresponding ALS values.

Cross-disease comparisons were made at two levels. At the gene level, log_2_ fold changes were compared by Spearman rank correlation over genes present in both comparisons, matched on symbol as required by the build difference noted in Section 4.1. Directional agreement was computed only among genes reaching adjusted *p* < 0.05 in both, and the number of such genes is reported alongside every agreement percentage. At gene-set level, normalised enrichment scores were compared by Spearman rank correlation across sets tested in both, together with the count of sets reaching FDR < 0.05 in both. All correlations are two-sided. Associations between categorical technical variables and diagnosis (Section 4.3) were tested by Pearson’s χ^2^ test.

Multiple testing is controlled by Benjamini–Hochberg within each differential expression comparison and by permutation FDR within each enrichment run. The ten ALS fits are nested and stratified views of one comparison rather than ten independent discoveries, and Benjamini–Hochberg was applied within each. Recomputed as a single family over all 215,200 gene-level tests, the primary model returns 2677 rather than 2692 genes in cervical cord and 2945 rather than 2955 in lumbar cord; every gene named in Section 2 keeps its call, and over the forty panel tests of Figure 2e, the axonal structure-and-transport panel remains significant in nine of ten fits and the regeneration-associated panel in none (Table S8). Panel tests, donor-level correlations, and χ^2^ diagnostics are reported uncorrected and are interpreted as directional summaries supporting the gene-set analysis of Section 4.7, not as independent confirmatory tests. Two properties of the panel test bound what it can support. The panel is a subset of the background against which it is tested, so the comparison is not between independent samples; it measures only whether a panel sits off the centre of the transcriptome-wide distribution. The panels are also literature-derived rather than pre-registered. Leave-one-out sensitivity to panel membership is reported only for the regeneration-associated panel, the one panel whose significance turns on membership; the axonal, complement and neuroinflammation results are carried by the gene-set analysis of Section 4.7 and by individual gene-level effect sizes rather than by the panel statistic.

### 4.9. Software and Versions

All analyses were performed in Python 3.11.10 with PyDESeq2 0.5.4 [42], Scanpy 1.11.5 [44], and AnnData 0.12.19 [45]. Enrichment used GSEApy 1.3.1 [49]; general numerical work used pandas 2.3.3 [60], NumPy 2.4.6 [61], SciPy 1.17.1, and statsmodels 0.14.6 [62]. Gene annotation throughout is GENCODE v30 [40], the same release used by the data generators to quantify GSE153960 (Section 4.1); that release file contains 58,870 genes, of which 19,986 are protein-coding.

## 5. Conclusions

Reanalysed under one set of rules, the ALS cord and PD midbrain datasets converge on two axes. Neither is the intrinsic axonal program that the shared-mechanism hypothesis predicts. The first is a complement and phagocytosis program that these data place in microglia. A signature defined in ALS cervical cord scores highest on microglia in all 11 donors of GSE157783 (+3.55, s.d. 0.536, against +0.73 for the next class). This test carries no diagnostic term, so it locates the transcripts rather than attributing them to disease. Within PD microglial pseudobulk, the same panel returns no increase (median Wald +0.21, *p* = 0.57, 5 PD, and 6 control donors). At this number of donors, that null cannot be distinguished from a larger microglial population. The second axis is a block of translation and ribosome gene sets containing the GCN2 response to amino acid deficiency. It is enriched in the ranked lists of both diseases. On the PD side, it is measured in a compartment whose composition differs between the groups compared, so it carries the same reservation as the axonal panel.

Microtubule-based axonal transport is the most depleted program in ALS cervical cord (normalised enrichment score −2.40, false discovery rate < 0.001; 155 ALS, 41 control). The ten-gene axonal structure-and-transport panel is depleted in nine of ten model fits. In bulk cord, that depletion is not separable from neuronal loss, which the pan-neuronal panel tracks at least as strongly. In PD dopaminergic neurons, the corresponding question is not adjudicable, because donor-level panel scores track dopaminergic content (ρ = −0.525, *p* = 0.031, *n* = 17 donors).

The contributing project is a second batch variable that any reuse of GSE153960 has to model. It tracks diagnosis in both cord segments analysed and separates it completely into three cortical regions. Adjusting for it removes about a third of the naive differential expression.

## Data Availability

The raw data reanalysed here are publicly available from the Gene Expression Omnibus under accessions GSE153960 (ALS bulk RNA-seq of spinal cord and cortex), GSE157783 (midbrain single-nucleus RNA-seq), and GSE178265 (substantia nigra single-nucleus RNA-seq). Raw human sequence for GSE178265 is under controlled access through dbGaP and was not used; this analysis relies exclusively on the public processed matrix distributed with the accession. Everything derived from those accessions in this study—the analysis and figure-rendering code, the processed per-panel source tables behind every figure, and the supplementary tables—is openly available at https://github.com/chaeyunjung/pd-als-transcriptomics (accessed on 28 August 2026). Tables S1–S7 are additionally provided as supplementary files with this article. A large language model was used to assist with drafting and editing the manuscript text and with writing the analysis and figure-rendering code; all analyses were specified, executed, and checked by the author, who takes full responsibility for the content.

## Supplementary Materials

The following supporting information can be downloaded at: https://www.mdpi.com/article/10.3390/ijms27188011/s1.

## Abbreviations

The following abbreviations are used in this manuscript: ALS, amyotrophic lateral sclerosis; CNS, central nervous system; DEG, differentially expressed gene; FACS, fluorescence-activated cell sorting; FDR, false discovery rate; GEO, Gene Expression Omnibus; ISR, integrated stress response; NES, normalised enrichment score; NYGC, New York Genome Center; PD, Parkinson’s disease; snRNA-seq, single-nucleus RNA sequencing; UMI, unique molecular identifier.
