# Supplementary material for: Batch- and Composition-Controlled Reanalysis of Bulk and Single-Nucleus Transcriptomes Reveals Co-Enrichment of Glial Complement and of Translational Signatures in Parkinson’s Disease and Amyotrophic Lateral Sclerosis
Source: Int J Mol Sci. 2026 Sep 9;27(18):8011. doi: 10.3390/ijms27188011 (PMC13606441; doi:10.3390/ijms27188011)
Supplement: Supplementary file 1 [file ijms-27-08011-s001.zip › Supplementary Materials.pdf]

International Journal of *Molecular Sciences*

MDPI

Supplementary Materials

Batch- and Composition-Controlled Reanalysis of Bulk and Single-Nucleus Transcriptomes Reveals Co-Enrichment of Glial Complement and of Translational Signatures in Parkinson's Disease and Amyotrophic Lateral Sclerosis

Chaeyun Jung 1*

1 Department of Experimental and Clinical Pharmacology, College of Pharmacy, University of Minnesota, Minneapolis, MN 55455, United States; jung0433@umn.edu

* Correspondence: jung0433@umn.edu

Contents

Figure S1. Library preparation, contributing project and donor structure of the ALS cohort.

Figure S2. The analyses excluded from the primary comparison, and the diagnostics on which they were excluded.

Figure S3. Effect of the neuronal-content covariate on marker genes, on curated panels and on the set of significant genes.

Figure S4. Cell-type differential expression, donor coverage and whole-tissue enrichment in the PD midbrain.

Figure S5. Residual structure of the two unintegrated embeddings, and two placement diagnostics that support no test.

Figure S6. Gene-set enrichment in PD microglia, and the cell-type distribution of the complement and phagocytosis genes.

Figure S7. Anatomical, provenance and marker characterisation of the dopaminergic-enriched selection.

Table S1. Sample-level metadata for the 1,639 GSE153960 libraries matched to the count matrix, with primary-analysis membership and exclusion reasons (1,639 rows).

Table S2. Composition of the curated gene panels and their availability in each of the three transcriptomic datasets (153 rows).

Table S3. Donor-level metadata for the dopaminergic-enriched nuclei selected from GSE178265 (18 rows).

Table S4. Complete differential-expression results for the primary model (M3) in ALS cervical and lumbar spinal cord (43,046 rows).

Table S5. The 326 gene sets reaching FDR < 0.05 in both the NYGC and the Target ALS cervical-cord stratum, with their enrichment in the primary model (326 rows).

Table S6. Per-cell-type differential-expression summary for PD versus control in GSE157783 midbrain (10 rows).

Table S7. The 86 gene sets significantly enriched in both ALS cervical spinal cord and PD dopaminergic neurons (86 rows).

Table S8. Robustness of the ALS analysis to joint multiple-testing correction, to the specification of the composition covariate, to a donor random effect, and to full covariate adjustment. Six sections: (A) differentially expressed gene counts under Benjamini-

Hochberg applied within each of the ten ALS fits and as one family over all 215,200 gene-level tests; (B) the forty panel tests of Figure 2e, uncorrected and under one BH family; (C) panel statistics under six composition-covariate specifications in both cord segments, with genome-wide agreement against the primary model; (D) donor random intercept against fixed effects in the joint two-segment model over 21,312 genes; (E) panel statistics recomputed on an independently published fully covariate-adjusted analysis of the same spinal cord samples [15]; (F) length-matched permutation of three panels. Delivered as TableS8_robustness_checks.tsv (236 rows).

Every table is supplied in full as a machine-readable tab-separated file, named under each table below. The first rows of each are reproduced here so that the columns can be read without opening the files.

Supplementary Figures

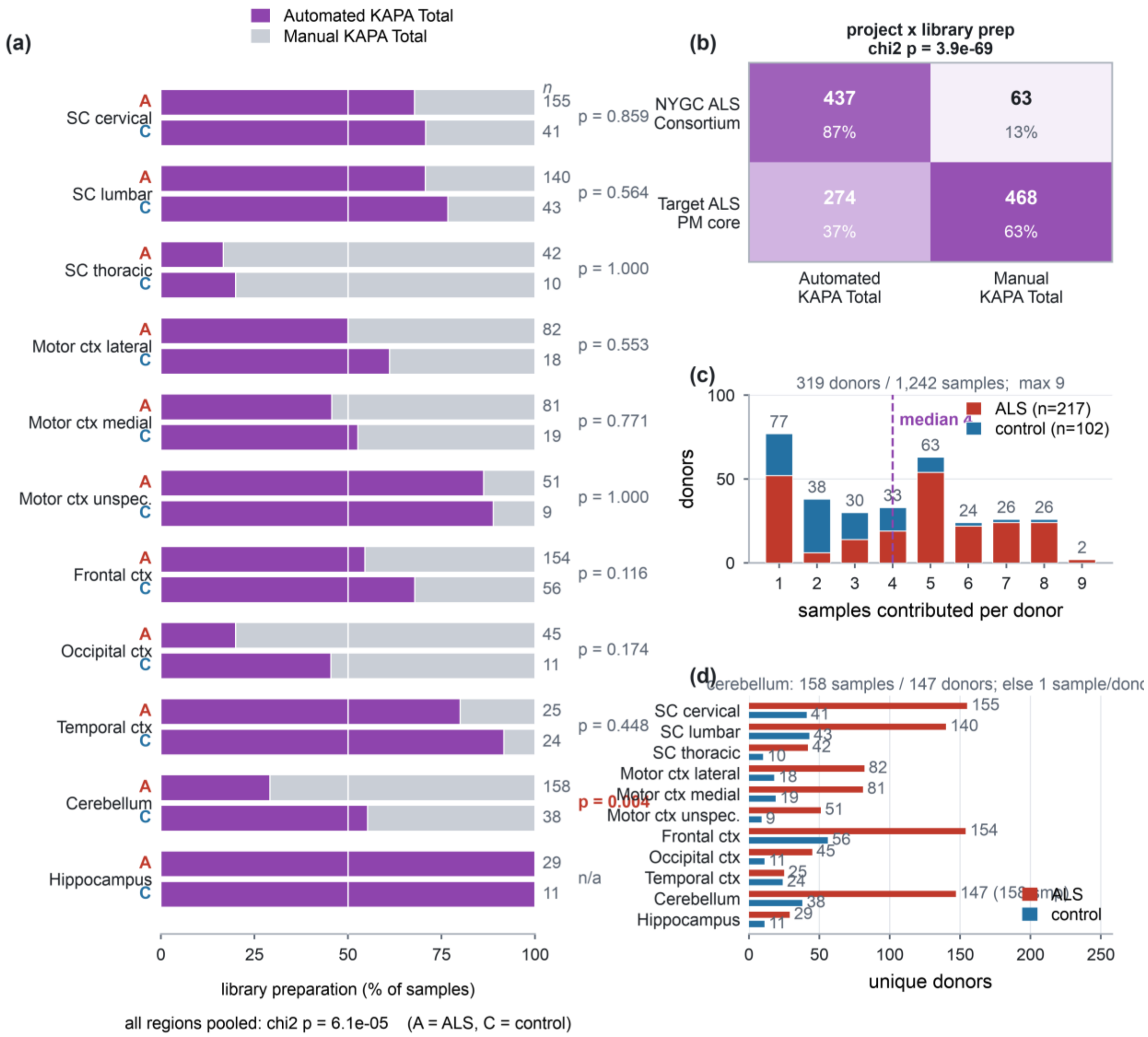

Figure S1. Library preparation, contributing project and donor structure of the ALS cohort. All panels describe the 1,242 GSE153960 samples of the primary analysis set — 962 ALS from 217 donors, 280 control from 102 donors [15,16]. (**a**) Library protocol as a percentage of each region and diagnosis group; A, ALS; C, control. Purple, Automated KAPA Total; grey, Manual KAPA Total; italic *n* at each bar end, samples in that group. *P*, two-sided Pearson χ^2 of protocol against diagnosis within region, uncorrected, red where $P < 0.05$ and n.a. for hippocampus, where one protocol covers both groups. The value below the panel, χ^2 $P = 6.1 \times 10^{-5}$, is the pooled test. (**b**) Contingency table of contributing project against protocol over the same samples; each cell prints its count and

row percentage; shading is proportional to the count. *P* above the table is a two-sided Pearson χ^2 of the 2 × 2 table. (**c**) Donors contributing 1 to 9 samples, stacked by diagnosis (red, ALS, n = 217; blue, control, n = 102); the number above each bar is the column total; the dashed purple line marks the median of 4 samples per donor. No test. (**d**) Unique donors per region and group (red, ALS; blue, control), counts at the bar ends. Every region except cerebellum contributes at most one sample per donor; cerebellum contributes 158 ALS samples from 147 donors. No test.

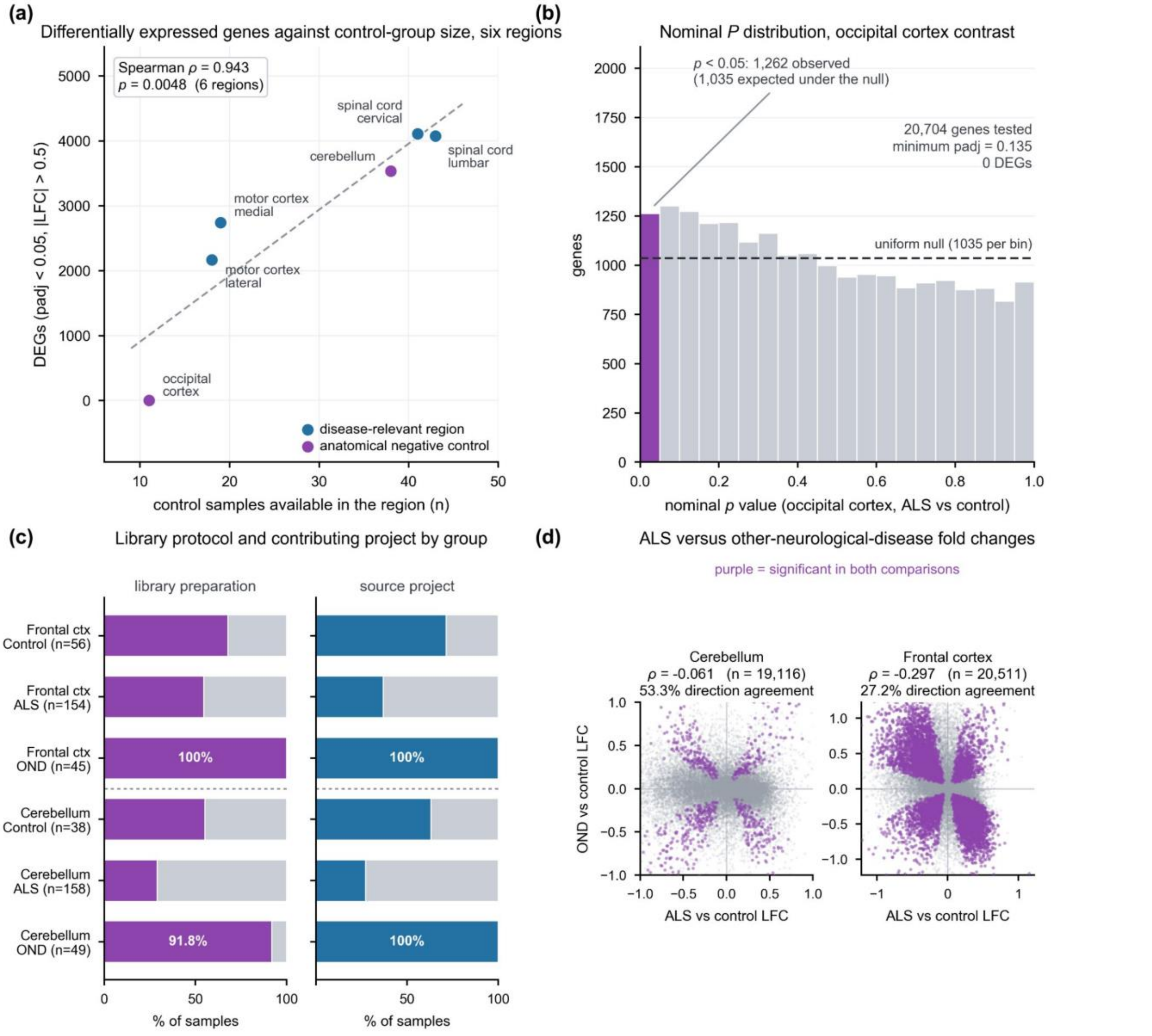

Figure S2. The analyses excluded from the primary comparison, and the diagnostics on which they were excluded. All panels describe GSE153960; **a** and **b** are fitted with ~ libprep + condition, not the primary model. (**a**) Genes at $P_{adj} < 0.05$ and $|\log_2 FC| > 0.5$ against the number of control samples available, one point per region for six regions. Blue, regions of disease relevance; purple, the two anatomical negative controls, cerebellum and occipital cortex; dashed line, a least-squares fit for orientation. The box gives the Spearman correlation over the six regions and its two-sided *P*. (**b**) Nominal *P* values from the occipital cortex contrast in 20 equal bins. The first bin, purple, holds 1,262 genes against 1,035 expected under a uniform null, marked by the dashed line. The 20,704 genes counted survive independent filtering and carry a *P* value; the summary underlying **a** counts 20,732 tested. The minimum Benjamini–Hochberg adjusted *P* is 0.135 and no gene is called. No test. (**c**) Library protocol (left) and contributing project (right) as a percentage of samples, for the control, ALS and other-neurological-disease (OND) groups of frontal cortex and cerebellum;

group sizes are in the axis labels. In-bar percentages mark groups drawn entirely or almost entirely from one level. No test. (**d**) Gene-wise $\log_2$ fold change of OND against control plotted against ALS against control, for cerebellum (left) and frontal cortex (right). Purple, genes at $P_{adj} < 0.05$ in both contrasts; grey, all others. Above each plot, ϱ is Spearman over genes quantified in both (*n* beside it) and the percentage is the share of doubly significant genes moving in the same direction.

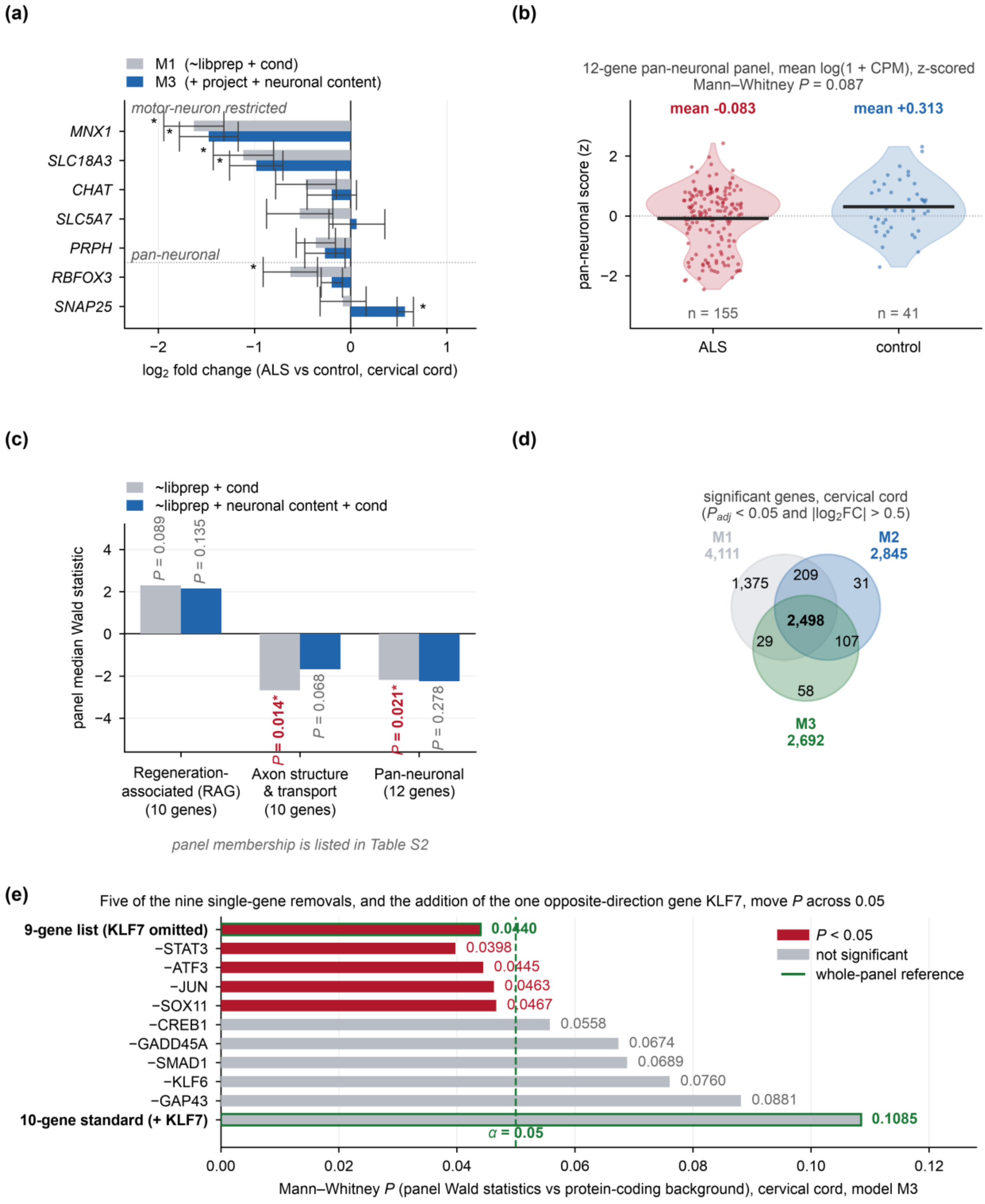

Figure S3. Effect of the neuronal-content covariate on marker genes, on curated panels and on the set of significant genes. All panels describe the cervical cord comparison of GSE153960, 155 ALS against 41 control, 21,506 genes tested, PyDESeq2 (Wald test, Benjamini–Hochberg adjustment). Models are M1, ~ libprep + condition; M2, ~ project + libprep + condition; M3, ~ project + libprep + neuro_score + condition. (**a**) $\log_2$ fold change of seven neuronal markers, the five motor-

neuron-restricted ones above the dotted rule and the two pan-neuronal ones below. Grey bars, M1; blue bars, M3; whiskers, ± 1 lfcSE. An asterisk marks $P_{adj} < 0.05$ for that gene under that model. (**b**) Neuronal-content proxy per sample: mean log(1 + CPM) over the twelve-gene pan-neuronal panel of Table S2, standardised within the region. One dot per sample (red, ALS, $n = 155$; blue, control, $n = 41$); violin, kernel density; heavy bar, group mean, printed above; dotted line, zero. *P* is a two-sided Mann–Whitney test between groups, uncorrected. (**c**) Median Wald statistic of three panels, tested before the neuronal-content covariate (grey, ~ libprep + condition) and after (blue, ~ libprep + neuro_score + condition). Neither fit carries project, so the blue bars are not M3. Panel sizes are 10, 10 and 12 genes; memberships in Table S2. Each *P* is a two-sided Mann–Whitney test against all protein-coding genes tested, uncorrected, red with an asterisk where $P < 0.05$. The pan-neuronal column is the covariate's own basis and not an independent control: ten of its twelve members build the covariate (pan_neuronal_composition_covariate_10 in Table S2). (**d**) Genes at $P_{adj} < 0.05$ and $|\log_2 FC| > 0.5$ under the three models as a Venn diagram; the number beside each label is that model's total, the number in each field the count in that intersection alone. No test. (**e**) Leave-one-out sensitivity of the regeneration-associated panel under M3. Each bar is the two-sided Mann–Whitney *P* of one variant against the protein-coding background, printed at the bar end; red, $P < 0.05$; grey, $P \geq 0.05$; dashed line, $\alpha = 0.05$. The two rows outlined in green are the reference memberships, the nine-gene list omitting *KLF7* and the full ten-gene panel; every other row drops one further gene.

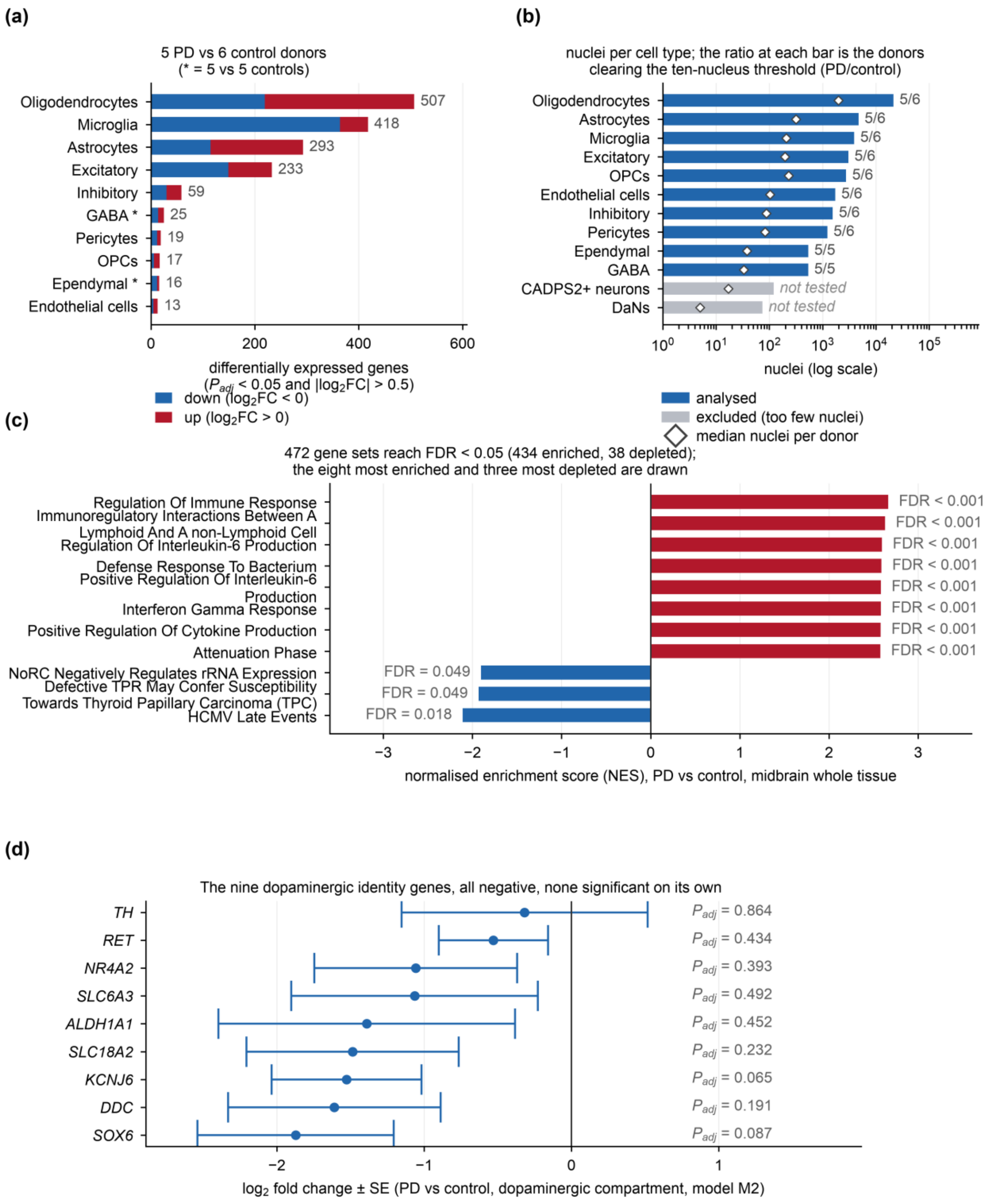

Figure S4. Cell-type differential expression, donor coverage and whole-tissue enrichment in the PD midbrain. Panels **a**–**c** describe donor pseudobulk of GSE157783 [17], 5 PD against 6 control donors; **d** describes the GSE178265 dopaminergic-neuron contrast, 9 disease against 8 control, under P2 (~ log(nuclei) + condition). (**a**) Genes at $P_{adj} < 0.05$ and $|log_2FC| > 0.5$ in each of the ten analysable cell types, stacked by direction (blue, lower in PD; red, higher), total at the bar end. An asterisk on a label marks the two classes for which only five of six control donors clear the ten-nucleus threshold; the two classes failing the donor thresholds do not appear. (**b**) Nuclei per annotated cell type on a logarithmic axis for all twelve classes: blue, analysed; grey, excluded for too few nuclei; open diamond, median nuclei per donor. The ratio at each bar end is the number of donors contributing at least ten nuclei, PD before control; excluded classes carry none. No test. (**c**) Normalised enrichment scores from pre-ranked gene-set enrichment analysis of whole-tissue pseudobulk, genes ranked by Wald statistic. Of the 472 sets at permutation FDR < 0.05 — 434

positive, 38 negative — the panel draws the eight highest and three lowest. Red, positive; blue, negative; FDR printed beside each bar and given as "FDR < 0.001" where the permutation estimate fell below that value. (**d**) $\log_2$ fold change of the nine dopaminergic identity genes; the circle is the point estimate and the whisker ± 1 lfcSE. The Benjamini–Hochberg adjusted *P* of each is printed at the right and none reaches 0.05, so no significance symbol is used.

https://doi.org/10.3390/xxxxx

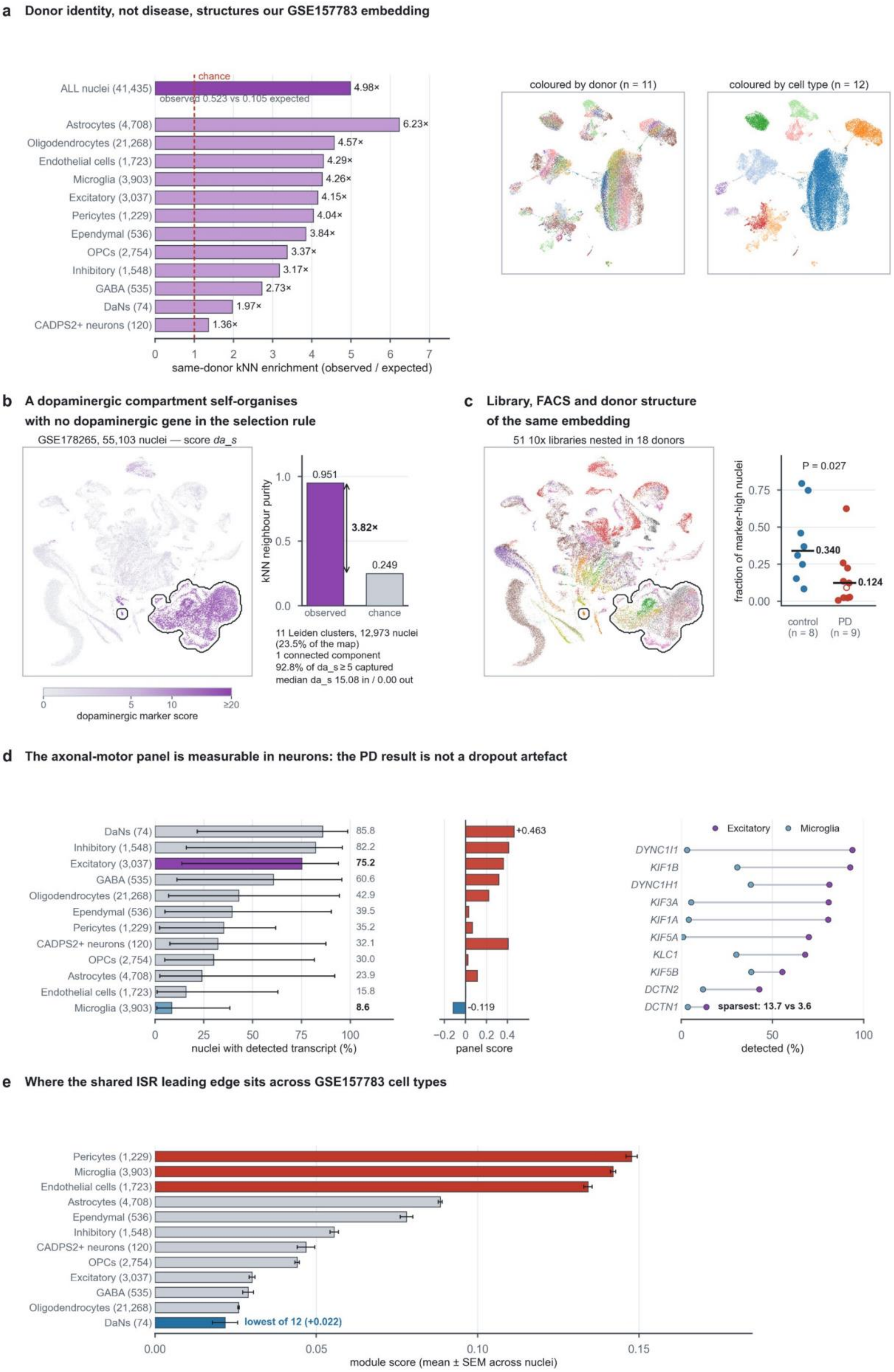

Figure S5. Residual structure of the two unintegrated embeddings, and two placement diagnostics that support no test. Neither embedding was donor- or batch-corrected, and neither is used to

compare diagnosis groups; the only group test in this figure is the donor-level comparison of marker-high fraction in panel (**c**). (**a**) Same-donor share among the 15 nearest neighbours of each nucleus in the GSE157783 embedding, divided by the share expected if donors were mixed at random within the same cell type, so 1.0 is chance and is marked by the red dashed line. The top bar is all 41,435 nuclei; the rest are the twelve cell types, nuclei in parentheses. Thumbnails at the right show the same embedding coloured by donor (11) and by the authors' annotation (12) [17]. No test. (**b**) The 55,103 selected GSE178265 nuclei on their own embedding, coloured by *da_s* (colour bar; values at or above 20 in one colour). The outline encloses the 11 Leiden clusters in which at least half the nuclei are marker-high — 12,973 nuclei, 23.5% of the selection — forming one connected component. Bars at the right give observed 15-nearest-neighbour purity against chance; the annotation below reports the cluster count, the share of marker-high nuclei captured and the median *da_s* inside and outside the outline. No test. (**c**) The same embedding coloured by donor as hue and library as tint, 51 libraries in 18 donors, the panel **b** compartment outlined; all 51 libraries are FACS *NR4A2*-positive, so the panel carries no FACS contrast. At the right, per-donor marker-high fraction by group (blue, control, $n = 8$; red, disease, $n = 9$); black bar, group median; open symbol, the donor excluded for contributing fewer than 100 nuclei. P is a two-sided donor-level Mann–Whitney test, uncorrected. (**d**) Percentage of nuclei with at least one count for each of the ten axonal transport motor genes across the twelve cell types. Left, the bar is the median over the ten genes and the whisker spans the gene-wise range — a range, not an interval estimate. Centre, the panel score per cell type, red at or above zero and blue below, with the highest and lowest printed. Right, the per-gene detection rate in excitatory neurons (purple) and microglia (blue), one connector per gene. (**e**) The recoverable part of the shared leading edge across the same twelve cell types, as the mean Scanpy module score of the 622 genes present here. Only the relative ordering is interpretable: 73 of the 695 genes are absent from this deposit, 61 of them cytoplasmic ribosomal proteins, so the absolute scores are not comparable with GSE178265 or with the ALS bulk data. Every cell type loses the same genes. Error bars are s.e.m. across nuclei and are pseudoreplicated, not a donor-level test; dopaminergic neurons rest on too few nuclei for their value to be precise. No test.

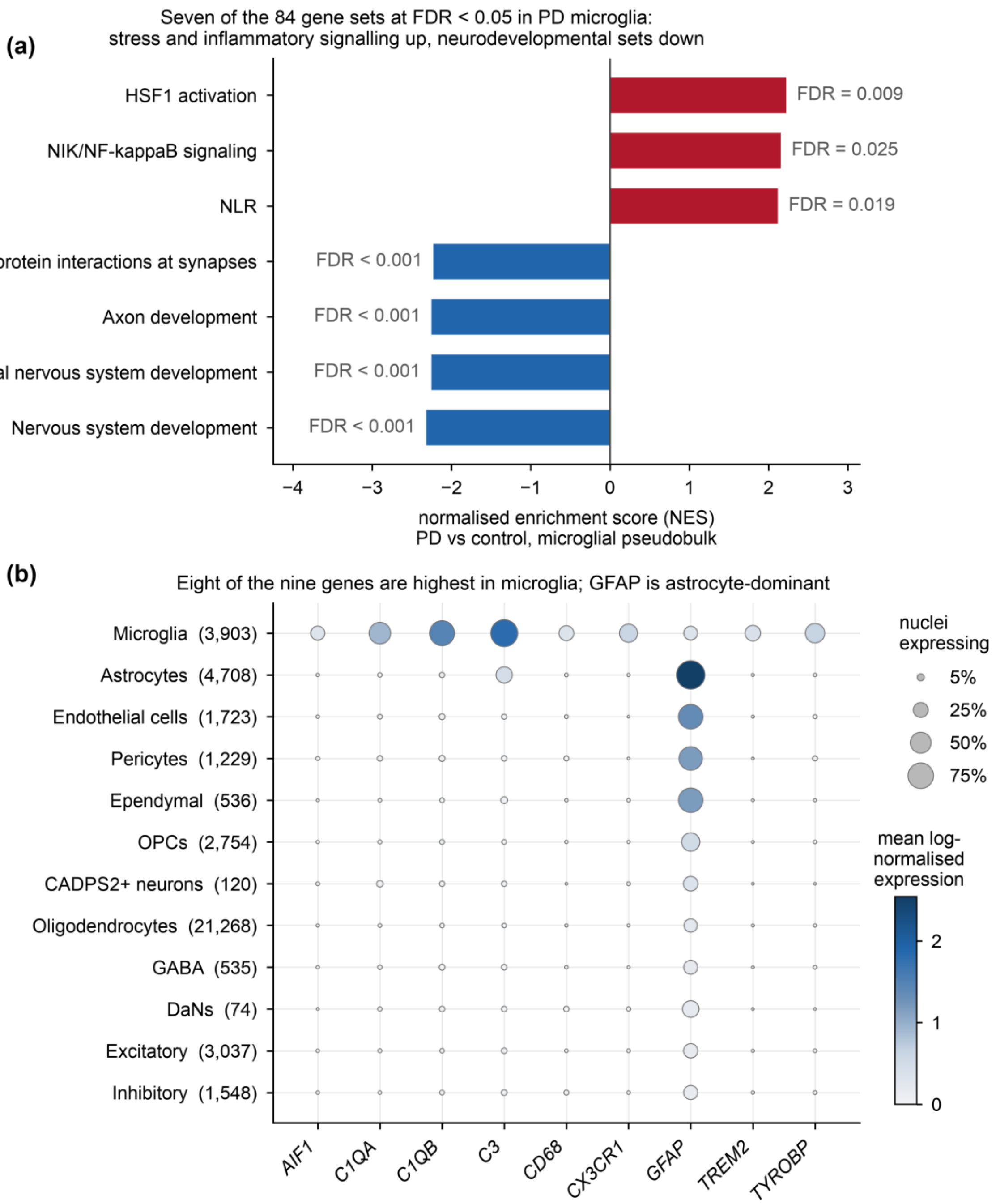

Figure S6. Gene-set enrichment in PD microglia, and the cell-type distribution of the complement and phagocytosis genes. Both panels describe GSE157783 [17], 41,435 nuclei from 5 PD and 6 control donors, under the authors' cell-type annotation. (**a**) Normalised enrichment scores (NES) from pre-ranked gene-set enrichment analysis of PD versus control in microglial donor pseudobulk, genes ranked by Wald statistic. Red, positive; blue, negative; vertical line, zero. Permutation FDR is printed beside each bar, as "FDR < 0.001" where the estimate fell below that value. Seven of the 84 sets at FDR < 0.05 are drawn — the four most negative and three of stress and inflammatory signalling — ordered by descending score. Names are shortened from Reactome 2022 and GO Biological Process 2023; NLR is Reactome NLR (inflammasome) signalling. (**b**) Expression of the nine genes of Figure 3f across the 12 cell types, PD and control nuclei pooled, so the panel shows cell-type distribution only and supports no comparison between diagnosis groups. Dot size, fraction of nuclei of that type with a non-zero count (key at right); dot colour, mean log-normalised expression over that type, scaled from zero to the panel maximum; nuclei per type in parentheses beside each row. Rows are ordered by the mean of the nine genes, a display order and not a test.

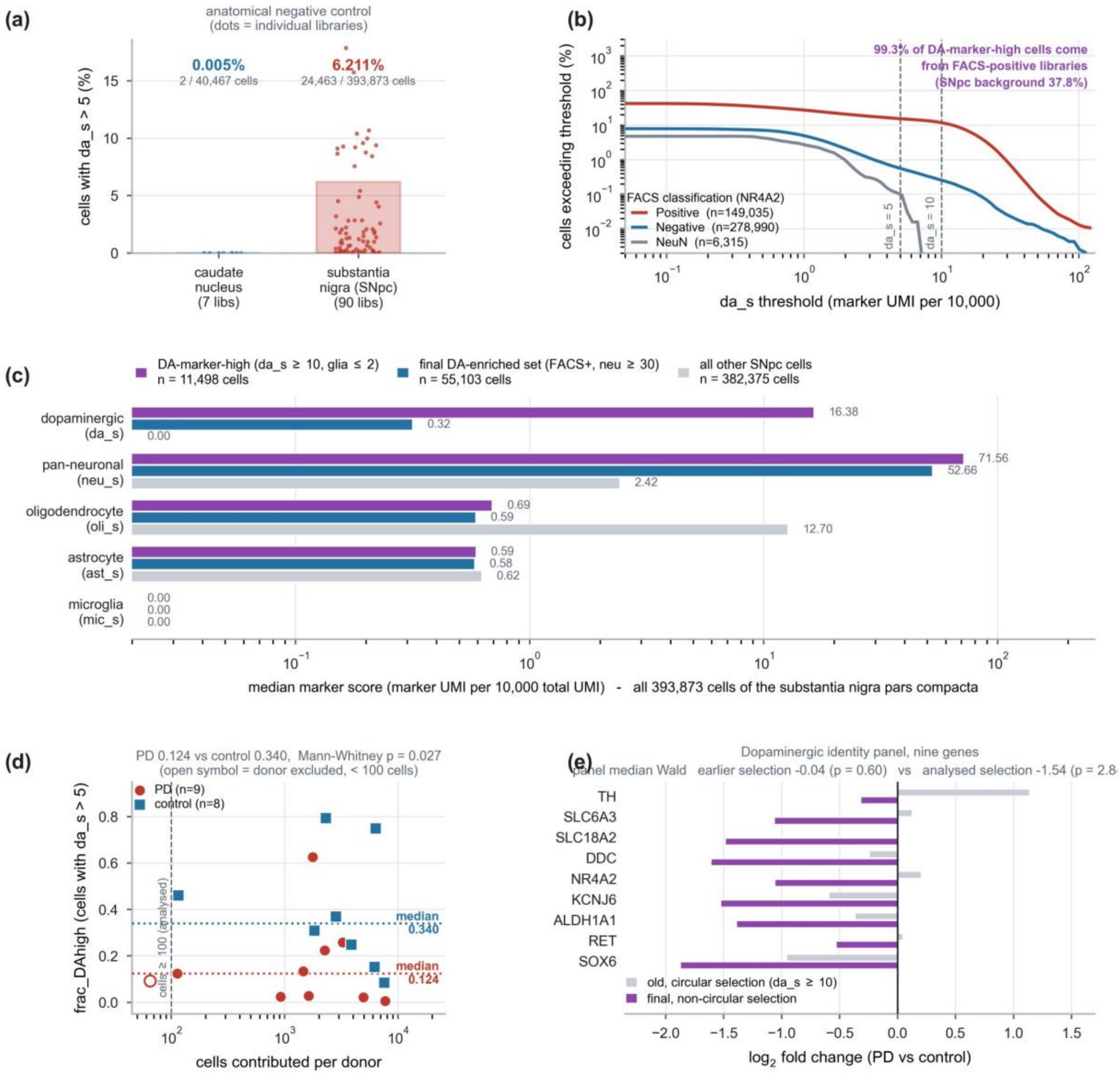

Figure S7. Anatomical, provenance and marker characterisation of the dopaminergic-enriched selection. All panels describe the public processed matrix of GSE178265 [18]. *da_s* is the summed *TH*, *SLC6A3*, *SLC18A2* and *DDC* count per 10,000 UMI; *neu_s*, *oli_s*, *ast_s* and *mic_s* are the corresponding pan-neuronal, oligodendrocyte, astrocyte and microglial scores (Table S2). (**a**) Percentage of nuclei with *da_s* > 5 in caudate nucleus (7 libraries, 40,467 nuclei) and substantia nigra pars compacta (90 libraries, 393,873 nuclei). Each dot is one library; the bar is the percentage pooled over the region, printed above with the underlying counts. No test. (**b**) Percentage of nuclei exceeding a sweep of *da_s* thresholds, one curve per recorded FACS classification: red, Positive (149,035 nuclei); blue, Negative (278,990); grey, NeuN (6,315). Both axes are logarithmic; dashed verticals mark *da_s* = 5 and 10. The annotation gives the share of the panel **c** marker-defined population arising from FACS-positive libraries, against 37.8% of all nigral nuclei. No test. (**c**) Median marker score, in marker UMI per 10,000 total UMI on a logarithmic axis, for three populations of the 393,873 nigral nuclei: purple, the marker-defined population (*da_s* ≥ 10, glial scores ≤ 2; *n* = 11,498), shown for comparison and entering no contrast; blue, the selection analysed here (FACS-positive, *neu_s* ≥ 30; *n* = 55,103); grey, the 382,375 nigral nuclei outside that marker-defined population — the complement of the purple group, not of the analysed selection, with which it overlaps. Values are printed at the bar ends; the microglial score is 0.00 in all three, so no bar is drawn there. No test. (**d**) Per-donor marker-high fraction against the nuclei that donor contributes (logarithmic axis). Red circles, disease; blue squares, control; filled, the 17 donors clearing the 100-nucleus floor (9 disease, 8 control) that enter the pseudobulk contrast; open symbol and dashed vertical, the one

https://doi.org/10.3390/xxxxx

donor below it. Dotted lines are group medians, printed at the right. *P* is a two-sided donor-level Mann–Whitney test over the 17 analysed donors, uncorrected. (**e**) $\log_2$ fold change, disease against control, of the nine dopaminergic identity genes. Purple, the selection analysed here; grey, the earlier marker-thresholded selection of panel **c**, for comparison only. The header prints each selection's median Wald statistic and its two-sided Mann–Whitney *P* against all protein-coding genes tested. Per-gene adjusted *P* values are in Figure S4d.

Supplementary Tables

Table S1. Sample-level metadata for the 1,639 GSE153960 libraries matched to the count matrix, with primary-analysis membership and exclusion reasons.

Columns: gsm, GEO sample accession; sample_id_alt, GEO characteristics field `sample id alt`; the join key to the count-matrix column name (`CGND-HRA-XXXXX`, with a `-2` suffix on re-sequenced libraries); tissue, Anatomical region as deposited; group, Normalised diagnostic group (identical to the raw deposited value for every row of this file); cond, Two-level condition used in the models: `ALS`, `Control`, or `NA` for samples outside the primary set; subject_id, Donor identifier; libprep, Library preparation protocol (`Automated KAPA Total` / `Manual KAPA Total`); project, Contributing project (`NYGC ALS Consortium` / `Target ALS PM core`); used_in_primary_analysis, TRUE for the 1,242 samples of the primary analysis set; exclusion_reason, Empty when used; otherwise the reason for exclusion.

gsm	sample_id_alt	tissue	group	cond	subject_id	libprep	project	used_in_primary_analysis	exclusion_reason
GSM4659178	CGND-HRA-00069	Cerebellum	ALS Spectrum MND	ALS	NEUEL133AK6	Manual KAPA Total	Target ALS PM core	TRUE	
GSM4659180	CGND-HRA-00071	Cerebellum	ALS Spectrum MND	ALS	NEUZU200WEQ	Manual KAPA Total	Target ALS PM core	TRUE	
GSM4659181	CGND-HRA-00073	Cerebellum	ALS Spectrum MND	ALS	NEUEC006FND	Manual KAPA Total	Target ALS PM core	TRUE	
GSM4659182	CGND-HRA-00075	Cerebellum	ALS Spectrum MND	ALS	NEUWX086DGZ	Manual KAPA Total	Target ALS PM core	TRUE	
GSM4660770	CGND-HRA-00076	Cerebellum	ALS Spectrum MND	ALS	NEUJZ255ZC5	Manual KAPA Total	Target ALS PM core	TRUE	
GSM4659246	CGND-HRA-00079	Cerebellum	ALS Spectrum MND	ALS	NEUYB686REL	Manual KAPA Total	Target ALS PM core	TRUE	

First 6 of 1,639 rows, shown so that the columns can be read. The complete table is supplied as the machine-readable file TableS1_GSE153960_samples.tsv.

Table S2. Composition of the curated gene panels and their availability in each of the three transcriptomic datasets.

Columns: panel, Panel identifier (see key below); gene_symbol, HGNC gene symbol as used in the analysis code; ensembl_versioned, GENCODE v30 versioned Ensembl gene id for that symbol (protein-coding entry preferred); in_GSE153960, TRUE if `ensembl_versioned` is a row of the GSE153960 RSEM count matrix; in_GSE157783, TRUE if the unversioned Ensembl id is in the GSE157783 deposited gene list; in_GSE178265, TRUE if `gene_symbol` is in the GSE178265 feature list (that matrix is symbol-native, hg19); notes, Free text; populated only where a gene is absent from a dataset, giving the reason.

panel	gene_symbol	ensembl_versioned	in_GSE153960	in_GSE157783	in_GSE178265	notes
axon_transport_motors	KIF5A	ENSG00000155980.12	TRUE	TRUE	TRUE	
axon_transport_motors	KIF5B	ENSG00000170759.11	TRUE	TRUE	TRUE	
axon_transport_motors	KIF1A	ENSG00000130294.16	TRUE	TRUE	TRUE	
axon_transport_motors	KIF1B	ENSG00000054523.17	TRUE	TRUE	TRUE	
axon_transport_motors	DCTN1	ENSG00000204843.12	TRUE	TRUE	TRUE	
axon_transport_motors	DCTN2	ENSG00000175203.15	TRUE	TRUE	TRUE	

First 6 of 153 rows, shown so that the columns can be read. The complete table is supplied as the machine-readable file TableS2_curated_gene_panels.tsv.

Table S3. Donor-level metadata for the dopaminergic-enriched nuclei selected from GSE178265.

Columns: donor, Donor identifier parsed from the library title; disease, `Disease` or `Ctrl` as deposited (GSE178265 does not separate PD from dementia with Lewy bodies); sex, age, pmi, Donor sex, age in years, post-mortem interval in hours; n_cells, Selected nuclei contributed by that donor; umi, Total UMI summed over that donor's selected nuclei; frac_DAhigh, Fraction of that donor's selected nuclei with dopaminergic marker score > 5 per 10,000 UMI; used, TRUE if `n_cells` ≥ 100, the inclusion rule for the pseudobulk model.

donor	disease	sex	age	pmi	n_cells	umi	frac_DAhigh	used
3298	Ctrl	Male	79	22.0	115	2309215	0.461	TRUE
3322	Ctrl	Female	90	22.0	7536	249174489	0.085	TRUE
3345	Ctrl	Female	82	12.9	2310	40369378	0.794	TRUE
3346	Ctrl	Female	91	10.0	6191	235880976	0.153	TRUE
3482	Ctrl	Female	79	14.0	3870	258146578	0.249	TRUE
4956	Ctrl	Female	92	23.3	6355	144036467	0.749	TRUE

First 6 of 18 rows, shown so that the columns can be read. The complete table is supplied as the machine-readable file TableS3_GSE178265_DA_donor_metadata.tsv.

Table S4. Complete differential-expression results for the primary model (M3) in ALS cervical and lumbar spinal cord.

Columns: region, `cervical` or `lumbar`; ensembl_versioned, GENCODE v30 versioned gene id; gene_symbol, GENCODE v30 symbol; `NA` where the id is not in the v30 map; baseMean, DESeq2 normalised mean expression; log2FoldChange, log$_2$ fold change, ALS relative to control; lfcSE, standard error of the log$_2$ fold change; stat, Wald statistic (also the GSEA ranking metric); pvalue, Wald test *P* value; padj, Benjamini–Hochberg adjusted *P* value; significant, TRUE if `padj` < 0.05 **and** \ |`log2FoldChange`\ | > 0.5 — the DEG definition used throughout the manuscript.

region	ensembl_versioned	gene_symbol	baseMean	log2FoldChange	lfcSE	stat	pvalue	padj	significant
cervical	ENSG00000000003.14	TSPAN6	357.463	0.566608	0.0832178	6.80874	9.84563e-12	2.36317e-10	TRUE
cervical	ENSG00000000419.12	DPM1	184.327	0.367253	0.0753683	4.87277	1.10044e-06	7.25031e-06	FALSE
cervical	ENSG00000000457.14	SCYL3	199.395	0.0391188	0.0535459	0.730565	0.465045	0.552861	FALSE
cervical	ENSG00000000460.17	C1orf112	142.797	-0.0241892	0.0559042	-0.43269	0.66524	0.735429	FALSE
cervical	ENSG00000000938.13	FGR	304.546	0.638223	0.158669	4.02236	5.76167e-05	0.000233134	TRUE
cervical	ENSG00000000971.15	CFH	971.121	0.0169347	0.177552	0.095379	0.924014	0.943275	FALSE

First 6 of 43,046 rows, shown so that the columns can be read. The complete table is supplied as the machine-readable file TableS4_ALS_M3_DEG_full.tsv.

Table S5. The 326 gene sets reaching FDR < 0.05 in both the NYGC and the Target ALS cervical-cord stratum, with their enrichment in the primary model.

Columns: term, Gene-set name as given by the Enrichr library; library, Library the retained record came from; NES_NYGC, FDR_NYGC, Normalised enrichment score and FDR *q* value in the NYGC stratum (63 ALS / 28 control); NES_Target, FDR_Target, The same in the Target ALS stratum (92 ALS / 13 control); NES_M3, FDR_M3, The same in the primary cervical model M3, for reference; same_direction, TRUE if `NES_NYGC` and `NES_Target` share a sign.

term	library	NES_NYGC	FDR_NYGC	NES_Target	FDR_Target	NES_M3	FDR_M3	same_direction
L13a-mediated Translational Silencing Of Ceruloplasmin	Reactome_2022	3.47197	0	2.40156	0	3.15401	0	TRUE

Expression R-HSA-156827								
SRP-dependent Cotranslational Protein Targeting To Membrane R-HSA-1799339	Reactome_2022	3.45931	0	2.39507	0	3.1216	0	TRUE
GTP Hydrolysis And Joining Of 60S Ribosomal Subunit R-HSA-72706	Reactome_2022	3.44534	0	2.3823	0	3.13988	0	TRUE
Viral mRNA Translation R-HSA-192823	Reactome_2022	3.41709	0	2.40765	0	3.07217	0	TRUE
Cap-dependent Translation Initiation R-HSA-72737	Reactome_2022	3.4106	0	2.38697	0	3.1099	0	TRUE
Formation Of A Pool Of Free 40S Subunits R-HSA-72689	Reactome_2022	3.40283	0	2.36219	0.000147122	3.06975	0	TRUE

First 6 of 326 rows, shown so that the columns can be read. The complete table is supplied as the machine-readable file TableS5_stratified_GSEA_shared326.tsv.

Table S6. Per-cell-type differential-expression summary for PD versus control in GSE157783 midbrain.

Columns: cell_type, Author cell-type annotation; n_donors_PD, PD donors entering the model for that cell type; n_donors_Control, Control donors entering the model for that cell type; n_nuclei, Total nuclei of that cell type in GSE157783; n_genes_tested, Genes surviving the low-expression filter; n_DEG, Genes with `padj` < 0.05 and \|log$_2$FC\| > 0.5; n_up, n_down, Direction split of `n_DEG`; min_padj, Smallest adjusted *P* value in that comparison.

cell_type	n_donors_PD	n_donors_Control	n_nuclei	n_genes_tested	n_DEG	n_up	n_down	min_padj
Oligodendrocytes	5	6	21268	17313	507	288	219	7.96949e-11
Microglia	5	6	3903	11215	418	54	364	0.000568413
Astrocytes	5	6	4708	13745	293	178	115	3.12569e-06
Excitatory	5	6	3037	16710	233	84	149	9.68727e-09
Inhibitory	5	6	1548	14812	59	29	30	7.05699e-13
GABA	5	5	535	8583	25	11	14	6.27957e-07

First 6 of 10 rows, shown so that the columns can be read. The complete table is supplied as the machine-readable file TableS6_PD_celltype_DEG_summary.tsv.

Table S7. The 86 gene sets significantly enriched in both ALS cervical spinal cord and PD dopaminergic neurons.

Table S8. Robustness of the ALS analysis to joint multiple-testing correction, to the specification of the composition covariate, to a donor random effect, and to full covariate adjustment. Six sections: (A) differentially expressed gene counts under Benjamini-Hochberg applied within each of the ten ALS fits and as one family over all 215,200 gene-level tests; (B) the forty panel tests of Figure 2e, uncorrected and under one BH family; (C) panel statistics under six composition-covariate specifications in both cord segments, with genome-wide agreement against the primary model; (D) donor random intercept against fixed effects in the joint two-segment model over 21,312 genes; (E) panel statistics recomputed on an independently published fully covariate-adjusted analysis of the same spinal cord samples [15]; (F) length-matched permutation of three panels. Delivered as TableS8_robustness_checks.tsv (236 rows).

Columns: term, Gene-set name; library, Library the retained record came from; NES_ALS_cervical, FDR_ALS, Enrichment in ALS cervical spinal cord, model M3;

NES_PD_DA, FDR_PD_DA, Enrichment in PD dopaminergic-enriched nuclei, model P2 (`~ log_cells + cond`); both_positive, TRUE if both normalised enrichment scores are positive.

term	library	NES_ALS_cervical	FDR_ALS	NES_PD_DA	FDR_PD_DA	both_positive
L13a-mediated Translational Silencing Of Ceruloplasmin Expression R-HSA-156827	Reactome_2022	3.15401	0	2.6946	0	TRUE
GTP Hydrolysis And Joining Of 60S Ribosomal Subunit R-HSA-72706	Reactome_2022	3.13988	0	2.70309	0	TRUE
SRP-dependent Cotranslational Protein Targeting To Membrane R-HSA-1799339	Reactome_2022	3.1216	0	2.52335	0	TRUE
Cap-dependent Translation Initiation R-HSA-72737	Reactome_2022	3.1099	0	2.65065	0	TRUE
Eukaryotic Translation Termination R-HSA-72764	Reactome_2022	3.08069	0	2.62682	0	TRUE
Viral mRNA Translation R-HSA-192823	Reactome_2022	3.07217	0	2.55015	0	TRUE

First 6 of 86 rows, shown so that the columns can be read. The complete table is supplied as the machine-readable file TableS7_shared_pathways_ALS_PD_DA.tsv.
